# The Type III Effectors NleE and NleB from Enteropathogenic *E. coli* and OspZ from *Shigella* Block Nuclear Translocation of NF-κB p65

**DOI:** 10.1371/journal.ppat.1000898

**Published:** 2010-05-13

**Authors:** Hayley J. Newton, Jaclyn S. Pearson, Luminita Badea, Michelle Kelly, Mark Lucas, Gavan Holloway, Kylie M. Wagstaff, Michelle A. Dunstone, Joan Sloan, James C. Whisstock, James B. Kaper, Roy M. Robins-Browne, David A. Jans, Gad Frankel, Alan D. Phillips, Barbara S. Coulson, Elizabeth L. Hartland

**Affiliations:** 1 Department of Microbiology and Immunology, University of Melbourne, Parkville, Victoria, Australia; 2 Centre for Pediatric Gastroenterology, UCL Medical School, Royal Free Campus, London, United Kingdom; 3 Department of Biochemistry and Molecular Biology, Monash University, Clayton, Victoria, Australia; 4 Department of Microbiology, Monash University, Clayton, Victoria, Australia; 5 ARC Centre for Structural and Functional Microbial Genomics, Monash University, Clayton, Victoria, Australia; 6 Centre for Vaccine Development and Department of Microbiology and Immunology, University of Maryland School of Medicine, Baltimore, Maryland, United States of America; 7 Centre for Molecular Microbiology and Infection, Division of Cell and Molecular Biology, Imperial College London, London, United Kingdom; Institut Pasteur, France

## Abstract

Many bacterial pathogens utilize a type III secretion system to deliver multiple effector proteins into host cells. Here we found that the type III effectors, NleE from enteropathogenic *E. coli* (EPEC) and OspZ from *Shigella*, blocked translocation of the p65 subunit of the transcription factor, NF-κB, to the host cell nucleus. NF-κB inhibition by NleE was associated with decreased IL-8 expression in EPEC-infected intestinal epithelial cells. Ectopically expressed NleE also blocked nuclear translocation of p65 and c-Rel, but not p50 or STAT1/2. NleE homologues from other attaching and effacing pathogens as well OspZ from *Shigella flexneri* 6 and *Shigella boydii*, also inhibited NF-κB activation and p65 nuclear import; however, a truncated form of OspZ from *S. flexneri* 2a that carries a 36 amino acid deletion at the C-terminus had no inhibitory activity. We determined that the C-termini of NleE and full length OspZ were functionally interchangeable and identified a six amino acid motif, IDSY(M/I)K, that was important for both NleE- and OspZ-mediated inhibition of NF-κB activity. We also established that NleB, encoded directly upstream from NleE, suppressed NF-κB activation. Whereas NleE inhibited both TNFα and IL-1β stimulated p65 nuclear translocation and IκB degradation, NleB inhibited the TNFα pathway only. Neither NleE nor NleB inhibited AP-1 activation, suggesting that the modulatory activity of the effectors was specific for NF-κB signaling. Overall our data show that EPEC and *Shigella* have evolved similar T3SS-dependent means to manipulate host inflammatory pathways by interfering with the activation of selected host transcriptional regulators.

## Author Summary

Bacterial intestinal pathogens have evolved distinct ways of colonizing the gut and causing disease. Enteropathogenic *E. coli* (EPEC) and its close relative enterohemorrhagic *E. coli* O157:H7 (EHEC) are extracellular pathogens that cause a characteristic lesion on the intestinal mucosa known as an attaching and effacing lesion. In contrast, *Shigella* is an intracellular pathogen that invades the intestinal mucosa and spreads from cell to cell. Both pathogens utilize a bacterial type III secretion system that “injects” virulence effector proteins into the host cell upon contact. We have discovered that an effector shared by EPEC/EHEC and *Shigella*, known as NleE or OspZ, as well as another EPEC/EHEC effector, NleB, inhibit the host cell inflammatory response by preventing translocation of the immune regulator NF-κB to the cell nucleus. Thus, although EPEC/EHEC and *Shigella* have evolved different colonization strategies, they share a common virulence determinant that suppresses the inflammatory response of the host, and both pathogens mediate a multi-effector attack on NF-κB signaling.

## Introduction

Many bacterial pathogens have the ability to “inject” virulence effector proteins into the host cell using a type III secretion system (T3SS). The effector proteins perform a variety of functions that allow the pathogen to persist in the host and cause disease [1]. Enteropathogenic *Escherichia coli* (EPEC) and enterohemorrhagic *E. coli* (EHEC) deliver T3SS effector proteins to the intestinal epithelium that mediate attaching and effacing lesion (A/E) lesion formation. A/E lesions are characterized by intimate bacterial attachment, effacement of the brush border microvilli and actin pedestal formation [2]. T3SS effectors from other pathogens such as *Salmonella* and *Shigella* have various roles in invasion, intracellular survival and the inhibition of innate immune responses through targeting host inflammatory signaling pathways [1]. Many of the T3SS effectors belong to conserved protein families that are found in a range of bacterial pathogens of plants and animals. For example, the OspF family of T3SS effectors from *Shigella*, *Salmonella* and *Pseudomonas* exhibit phosphothreonine lyase activity and induce irreversible dephosphorylation of mitogen-activated protein kinases (MAPKs) in the host cell nucleus [3], [4], [5]. In *Shigella*, this leads to gene-specific repression of a subset of NF-κB regulated genes, including *IL8* [3]. Given the remarkable specificity of their biochemical function, the discovery of the mechanism of action of T3SS effectors remains an important step towards understanding the pathogenesis of many bacterial infections.

The activation of gene expression during inflammation is tightly regulated by transcription factors such as NF-κB. The NF-κB/Rel family comprises five members that share an N-terminal Rel homology domain that mediates DNA binding, dimerization and nuclear translocation [6]. The p65, c-Rel and RelB NF-κB subunits have an additional C-terminal transactivation domain, which strongly activates transcription from NF-κB-binding sites in target genes. The p50 and p52 subunits lack the transactivation domain but still bind to NF-κB consensus sites and act as transcriptional repressors [6]. The most abundant form of NF-κB in mammalian tissues is a p65/p50 dimer that activates the expression of multiple cytokine genes in response to inflammatory signals. In resting cells, NF-κB subunits are held in an inactive form in the cytoplasm by binding IκB proteins. Activation of NF-κB signaling stimulates the phosphorylation and proteosomal degradation of IκB, whereupon the NF-κB dimer is transported into the nucleus through the nuclear pore complex [6]. The canonical NF-κB pathway is stimulated by a range cell surface receptors such as the TNF receptor, IL-1 receptor, Toll-like receptors and T-cell receptor. Although the upstream components of these pathways vary, they converge at the point of IκB kinase complex (IKK)-mediated phosphorylation of and subsequent degradation of IκB [7].

Enteropathogenic *Escherichia coli* (EPEC) and enterohemorrhagic *E. coli* (EHEC) utilize a type III secretion system (T3SS) to deliver effector proteins to the intestinal epithelium that induce actin pedestal formation [2]. Multiple additional effectors are transported into the host cell where their targets and effects on host cell biology remain largely uncharacterized [8]. NleE is a highly conserved 27 kDa T3SS effector protein of A/E pathogens encoded in an operon with the 38 kDa effector, NleB. The NleE homologue in the invasive pathogen, *Shigella*, is called OspZ [9], [10]. While investigating the effect of EPEC infection on NF-κB activation, we observed that wild type EPEC prevented translocation of the p65 subunit of NF-κB to the host cell nucleus, whereas an *nleE* mutant was defective for this activity. Here we report that NleE inhibits p65 nuclear translocation, thereby reducing the IL-8 response during bacterial infection, and that OspZ shares this activity. In addition, we show that NleB suppresses NF-κB activation but appears to act in distinct manner to NleE.

## Results

### EPEC infection blocks NF-κB p65 nuclear translocation, leading to reduced IL-8 production

**Figure 1:**
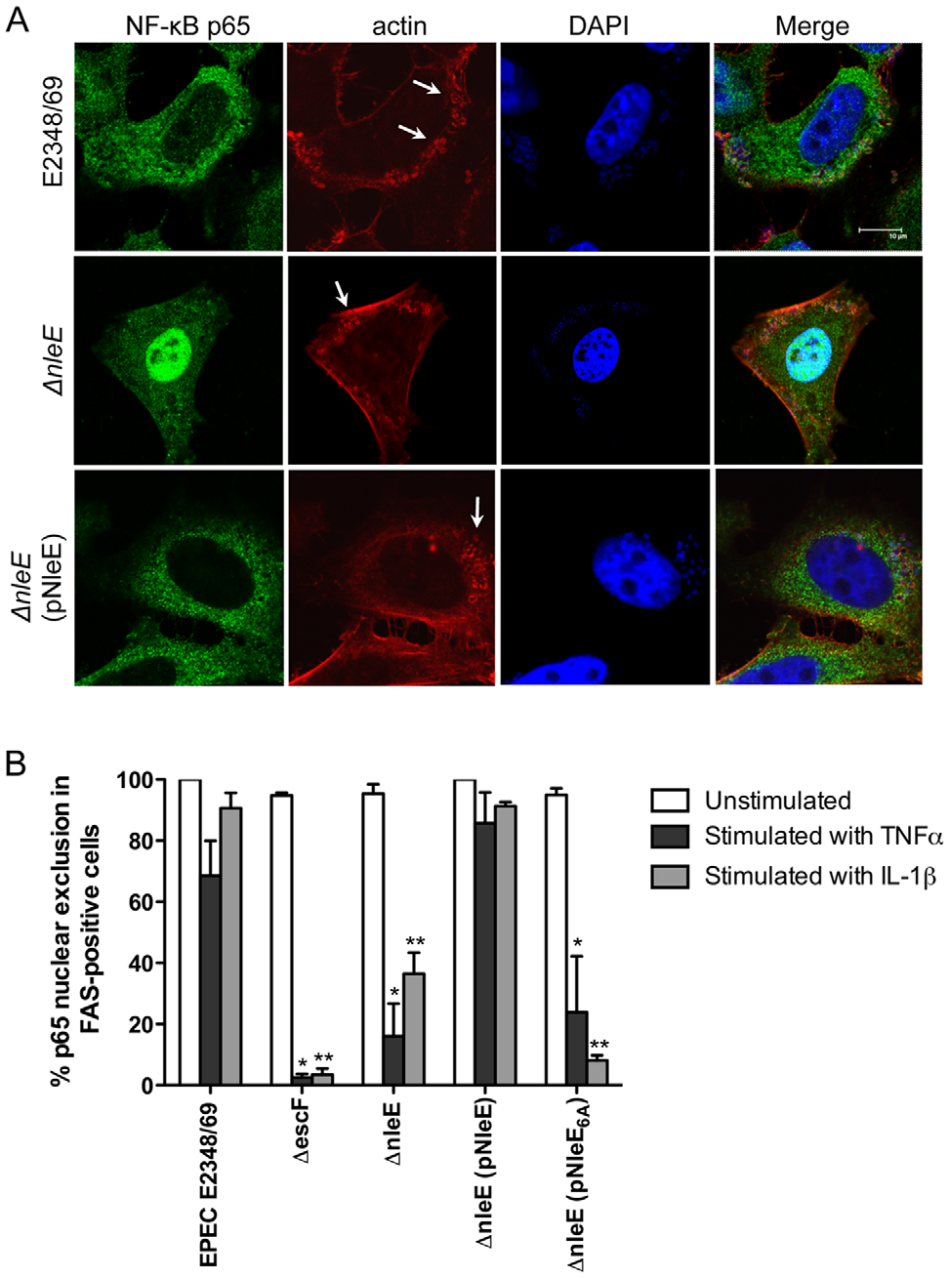
Effect of EPEC infection on NF-κB activation. **A**. Representative immunofluorescence fields showing p65 staining (green) in FAS-positive HeLa cells (red) infected with derivatives of EPEC E2348/69, stimulated with TNFα and stained for nucleic acid with DAPI (blue). Arrows indicate FAS-positive lesions. **B**. Quantification of p65 nuclear exclusion in cells infected with derivatives of EPEC E2348/69 and stimulated with TNFα or IL-1β. Results are expressed as the percentage of FAS-positive cells that exclude p65 from the nucleus and are the mean ± SEM of three independent experiments performed in duplicate. At least 100 FAS-positive cells were counted per test. *significantly different to E2348/69 stimulated with TNFα **significantly different to E2348/69 stimulated with IL-1β (*P*<0.05, one way ANOVA).

Recent work has shown that EPEC and EHEC infection inhibits inflammatory cytokine production and NF-κB activation [11], [12], [13]. Previously, we found that translocated NleE localised to the host cell nucleus and we postulated that NleE had a role in subverting innate immune signaling [10]. Here we investigated the effect of NleE on NF-κB activation during EPEC infection. As actin accumulation beneath adherent EPEC depends on successful translocation of the T3SS effector, Tir [2], we used the fluorescent actin staining (FAS) test as a general marker for the translocation of T3SS effectors. HeLa cells were infected with wild type EPEC E2348/69, a T3SS (*escF*) mutant, an *nleE* deletion mutant of EPEC or an *nleE* mutant complemented with full length *nleE*. Cell monolayers were either infected for 4 h and left unstimulated or infected for 90 min and stimulated with tumour necrosis factor α (TNFα) or interleukin-1β (IL-1β) for 30 min. Nuclear translocation of the p65 NF-κB subunit was visualised by immunofluorescence microscopy of FAS-positive cells for EPEC E2348/69, Δ*nleE* and Δ*nleE* (pNleE) (Fig. 1A) and cells with adherent bacteria for Δ*escF*. In unstimulated cells, there was no significant difference in p65 nuclear exclusion between wild-type infected cells and the *escF* mutant (Fig. 1B). In contrast, in cells stimulated with TNFα or IL-1β, wild type EPEC E2348/69 inhibited p65 transport to the nucleus, whereas the *escF* mutant had little inhibitory effect on p65 nuclear translocation (Fig. 1B). The *nleE* mutant also showed greatly reduced inhibition of p65 nuclear transport in response to TNFα or IL-1β compared to wild type EPEC E2348/69 which was restored upon complementation of the *nleE* mutant with a copy of full length *nleE*. Similar results were obtained in response to IL-1β in Caco-2 intestinal epithelial cells (Fig. S1).

**Figure 2:**
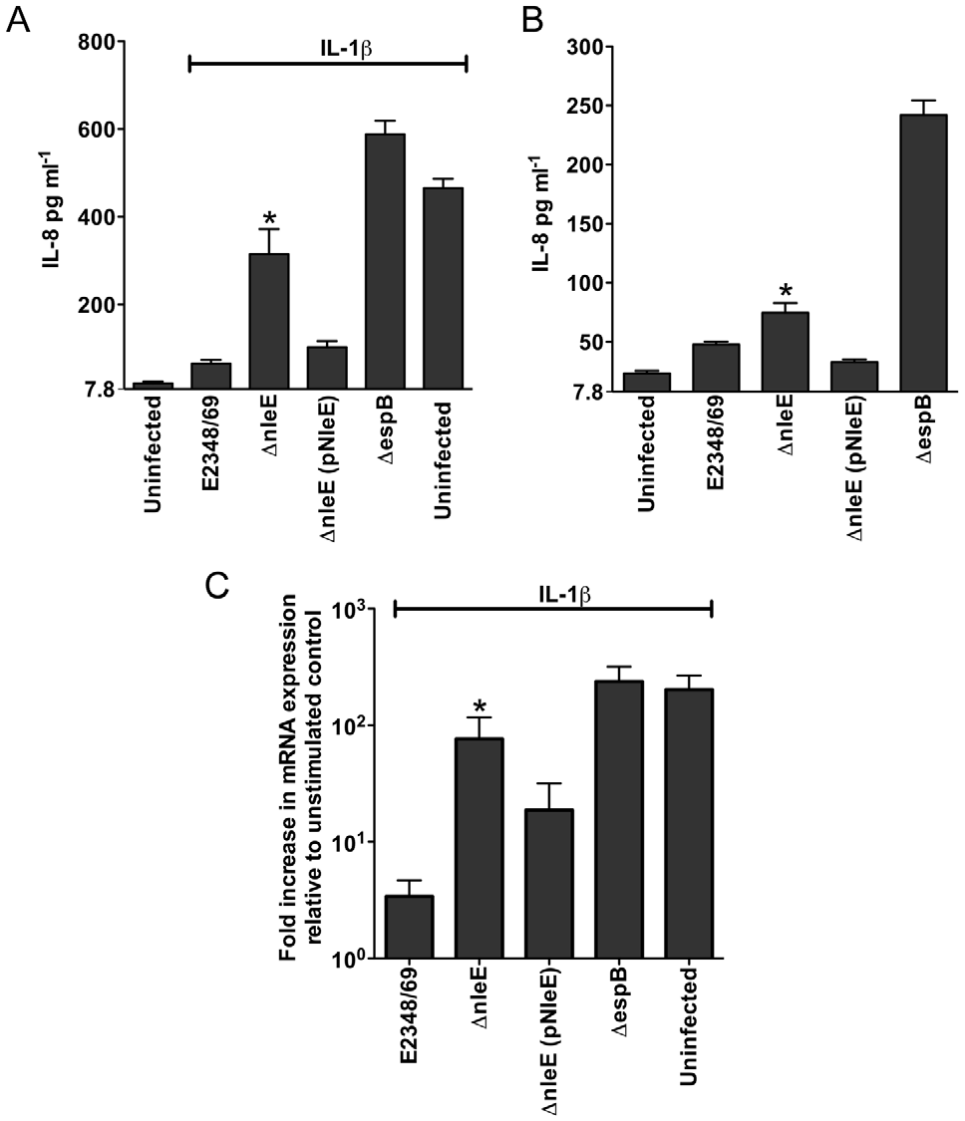
Effect of EPEC infection on IL-8 production and expression. **A**. IL-8 production from Caco-2 cells infected with derivatives of EPEC E2348/69 for 4 h and stimulated with IL-1β for 24 h. Results are the mean ± SEM of 5 independent experiments carried out in triplicate. *Δ*nleE* significantly different to E2348/69 and Δ*nleE* (pNleE) (*P*<0.05, one way ANOVA) **B**. IL-8 production from Caco-2 cells infected with derivatives of EPEC E2348/69 for 4 h and left unstimulated. Results are the mean ± SEM of 5 independent experiments carried out in triplicate. *Δ*nleE* significantly different to Δ*nleE* (pNleE) (*P*<0.05, one way ANOVA) **C**. Reverse transcription qPCR analysis of *IL8* expression in Caco-2 cells 4 h after infection and 3 h after IL-1β treatment. Results are expressed as log_10_ fold increase in mRNA expression over unstimulated control and are the mean ± SEM of 3 independent experiments carried out in triplicate. *Δ*nleE* significantly different to E2348/69 and Δ*nleE* (pNleE) (*P*<0.05, one way ANOVA).

Caco-2 intestinal epithelial cells were then utilised to determine if the inhibition of p65 translocation resulted in the suppression of IL-8 production. Caco-2 cells were incubated with wild type EPEC E2348/69, a T3SS (*espB*) mutant, the *nleE* mutant or the *nleE* mutant complemented with *nleE*. Cells were then stimulated with IL-1β [14]. Compared to infection with the *espB* mutant, wild type EPEC inhibited IL-8 production from Caco-2 cells. The *nleE* mutant showed a diminished capacity to inhibit IL-8 production, which was complemented to wild type levels upon reintroduction of full length *nleE* (Fig. 2A). A similar trend was observed in CaCo-2 cells left unstimulated, although the differences were not as great as in IL-1β-stimulated cells (Fig. 2B). Real time PCR analysis of *IL8* from Caco-2 cells infected with derivatives of EPEC and stimulated with IL-1β, showed that levels of *IL8* mRNA were suppressed by NleE expression (Fig. 2C).

### NleE inhibits nuclear translocation of p65 and c-Rel but not p50 or STAT1/2

**Figure 3:**
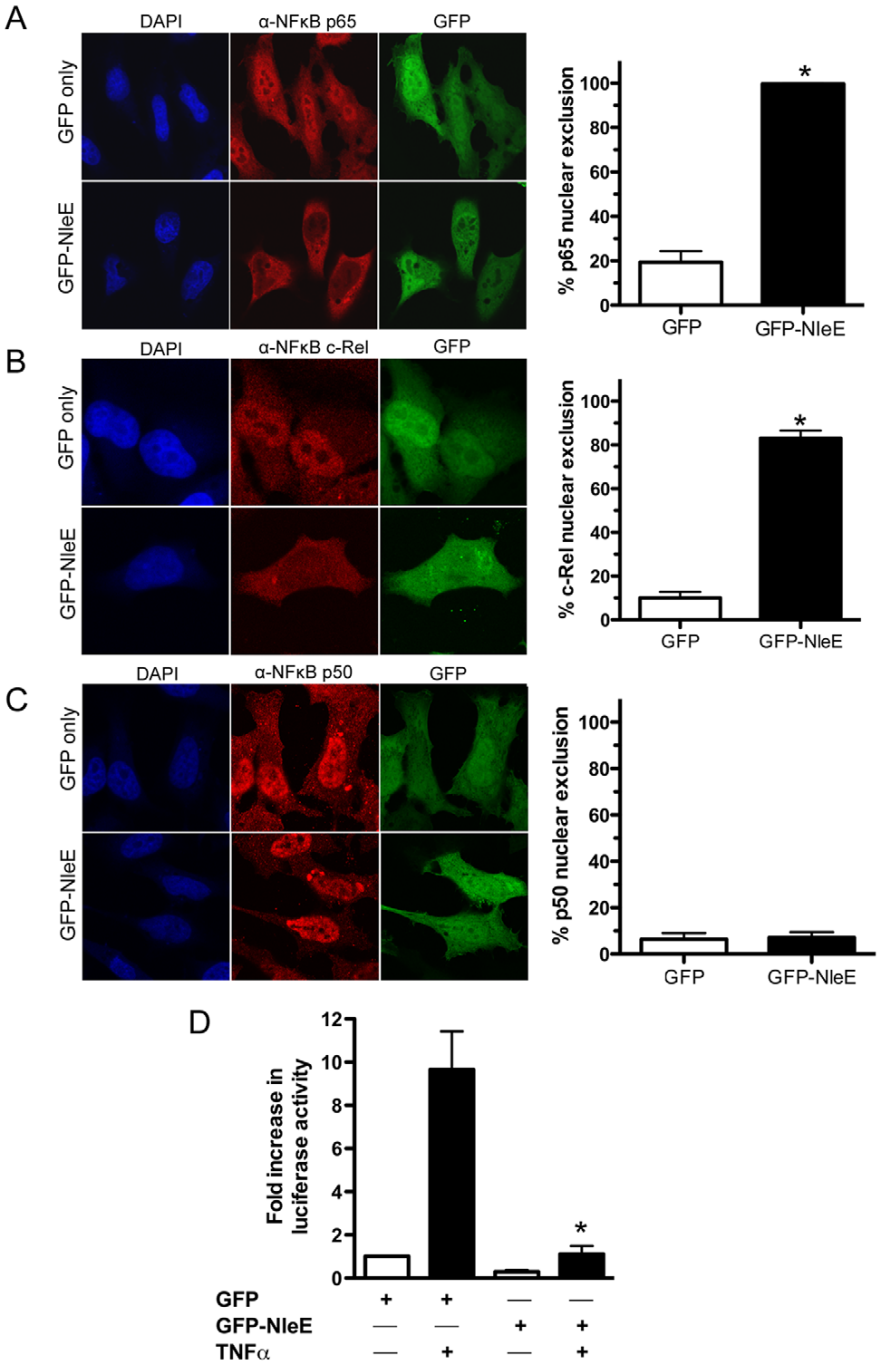
Effect of ectopically expressed NleE on NF-κB activation. **A**. Representative immunofluorescence fields and quantification of p65 nuclear exclusion using anti-p65 (**A**), anti-c-Rel (**B**) or anti-p50 antibodies (**C**) (red) of HeLa cells transfected with pEGFP-C2 (GFP only) or pGFP-NleE (green), stimulated with TNFα for 30 min, and stained for nucleic acid (blue). Results are expressed as the percentage of GFP-positive cells that exclude p65, c-Rel or p50 and are the mean ± SEM of three independent experiments performed in duplicate. At least 100 GFP-positive cells were counted per test. *significantly different to GFP only stimulated with TNFα, *P* = 0.0005 (unpaired, two-tailed *t*-test) **D**. Fold increase in NF-κB dependent luciferase activity in pEGFP-C2 (GFP) or pGFP-NleE transfected cells unstimulated (white bars) or stimulated with TNFα for 30 min (black bars). Results are the mean ± SEM of triplicate wells. *significantly different to GFP only stimulated with TNFα (*P*<0.05, one way ANOVA).

To determine if NleE was sufficient for the inhibition of p65 nuclear translocation, we expressed GFP-NleE or GFP transiently in HeLa cells. Upon stimulation with TNFα, the NF-κB p65 subunit was excluded from the nucleus in the presence of ectopically expressed GFP-NleE but not GFP alone (Fig. 3A). To ensure that this effect was not an artefact arising from the over expression of GFP-NleE, we examined a range of transfected cells exhibiting low, moderate and high GFP expression. Even in cells exhibiting low levels of GFP-NleE, p65 was excluded from the nucleus upon stimulation with TNFα (Fig. 3A and data not shown). We also investigated the effect of NleE on nuclear localization of other NF-κB proteins, c-Rel and p50. Similar to p65, NleE blocked nuclear translocation of c-Rel in response to TNFα (Fig. 3B), however p50 nuclear localization was unaffected by the presence of NleE (Fig. 3C). A dual-luciferase reporter system measuring the activation of NF-κB-dependent transcription confirmed the absence of NF-κB p65 nuclear activity in GFP-NleE transfected cells stimulated with TNFα (Fig. 3D).

**Figure 4:**
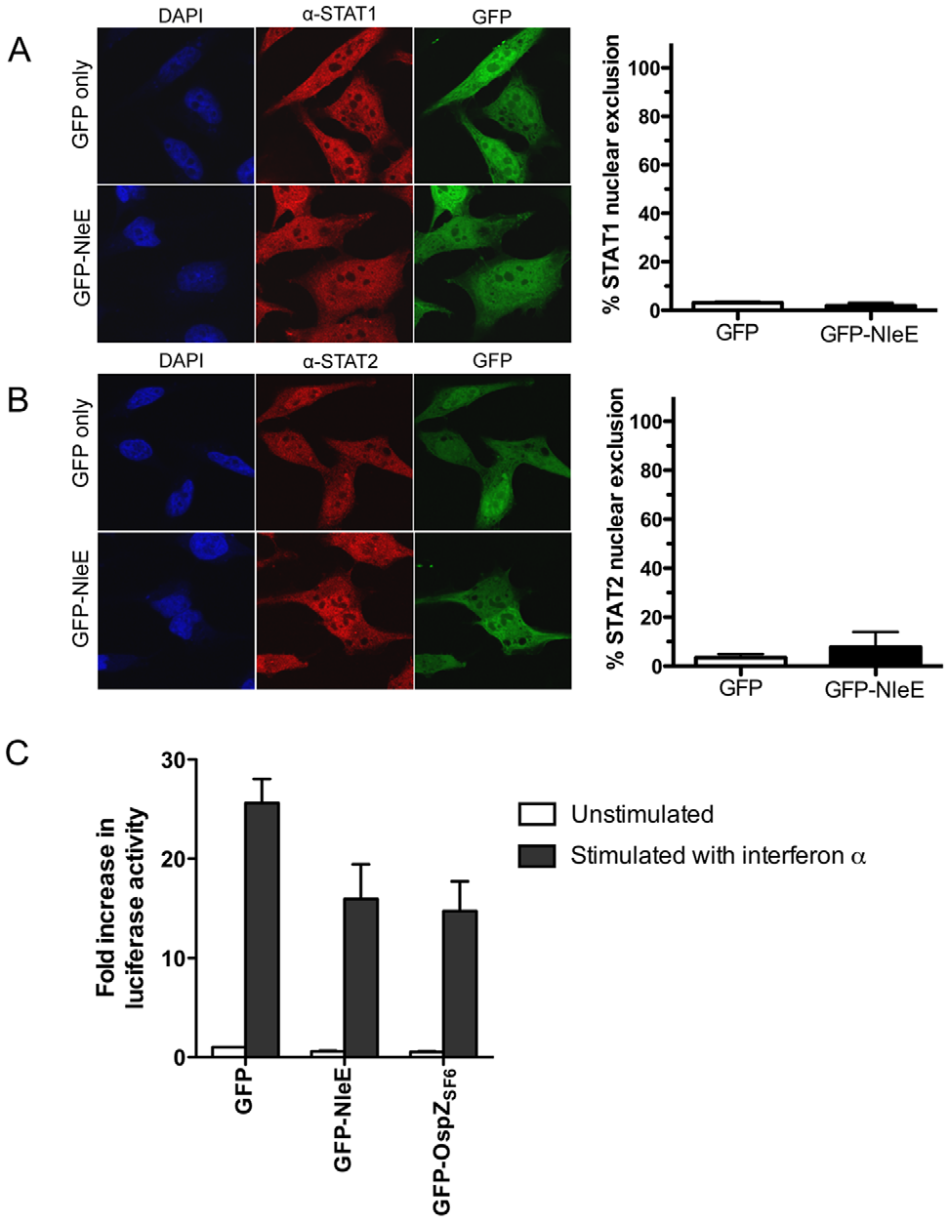
Effect of NleE on nuclear translocation of STAT1 and STAT2. **A**. Representative immunofluorescence fields and quantification of nuclear exclusion using antibodies to STAT1 (**A**) or STAT2 (**B**) (red) of HeLa cells transfected with pEGFP-C2 (GFP only) or pGFP-NleE (green), stimulated with IFNα for 30 min and stained for nucleic acid with DAPI (blue). Results are expressed as the percentage of GFP-positive cells that exclude STAT1 or STAT2 and are the mean ± SEM of three independent experiments performed in duplicate. At least 100 GFP-positive cells were counted per test. **C**. Fold increase in STAT1/2 dependent luciferase activity in HeLa cells transfected with pEGFP-C2 (GFP), pGFP-NleE or pGFP-OspZ. Results are the mean ± SEM of 3 independent experiments performed in triplicate. Differences between GFP and GFP-NleE or GFP-OspZ were not significant (*P*>0.05, one way ANOVA).

To determine if NleE-mediated inhibition of signaling affected other transcription factors, we tested the ability of NleE to inhibit nuclear translocation and activation of STAT1 and STAT2. Following stimulation with interferon α, both STAT1 and STAT2 were translocated to the nucleus in the presence of GFP or GFP-NleE (Fig. 4A and B). NleE also had no impact on STAT1/2 activation using a ISRE-Luc luciferase reporter (Fig. 4C) [15]. This suggests NleE acts on a subset of signaling pathways that includes NF-κB but not STAT1/2.

### NleE from other A/E pathogens and full length OspZ from *Shigella* inhibit p65 translocation

**Figure 5:**
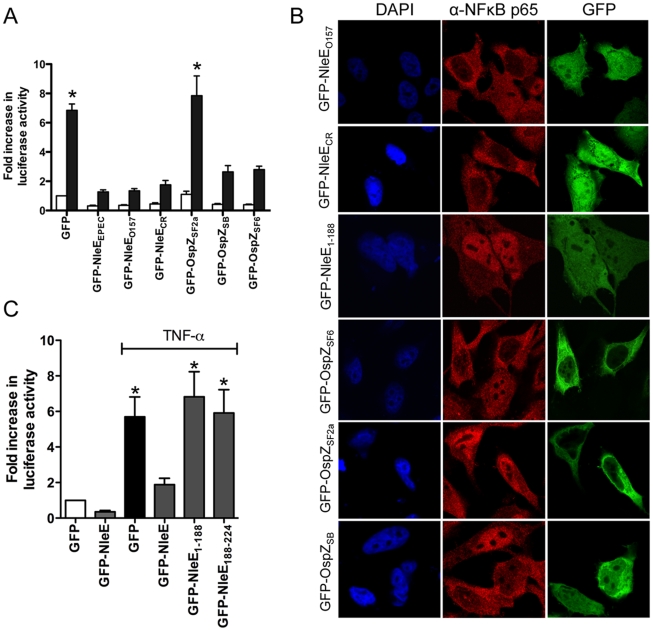
Effect of NleE and OspZ homologues on NF-κB activation. **A**. Fold increase in NF-κB dependent luciferase activity in HeLa cells transfected with pEGFP-C2 (GFP) or pGFP-NleE/OspZ cloned from *C. rodentium* (GFP-NleE_CR_), EHEC O157:H7 EDL933 (GFP-NleE_O157_), EPEC E2348/69 (GFP-NleE_EPEC_), *S. flexneri* 2a (GFP-OspZ_SF2a_), *S. boydii* (GFP-OspZ_SB_) and *S. flexneri* 6 (GFP-OspZ_SF6_) and left unstimulated (white bars) or stimulated with TNFα for 30 min (black bars). Results are the mean ± SEM of 3 independent experiments performed in triplicate. *significantly different to GFP-NleE_EPEC_ (*P*<0.05, one way ANOVA) **B**. Representative immunofluorescence fields using anti-p65 antibodies (red) of HeLa cells transfected with derivatives of pGFP-NleE and GFP-OspZ (green) labelled as in Fig. 5A and stimulated with TNFα for 30 min. **C**. Fold increase in NF-κB dependent luciferase activity in HeLa cells transfected with pEGFP-C2 (GFP) or pGFP-NleE, pGFP-NleE_1-188_ or pGFP-NleE_188-224_ and stimulated with TNFα for 30 min where indicated. Results are the mean ± SEM of 3 independent experiments performed in triplicate. *significantly different to GFP-NleE stimulated with TNFα (*P*<0.05, one way ANOVA).

To determine if the function of NleE and OspZ was conserved across A/E pathogens and *Shigella*, GFP-NleE and GFP-OspZ fusions generated from enterohemorrhagic *E. coli* O157:H7, *Citrobacter rodentium*, *Shigella boydii* and *Shigella flexneri* were expressed by transfection in HeLa cells. NleE from EHEC O157:H7 and *C. rodentium*, and full length OspZ from *S. boydii* and *S. flexneri* serogroup 6 inhibited NF-κB activation and p65 nuclear translocation (Fig. 5A and B). In contrast, OspZ from *S. flexneri* serogroup 2a which carries a 36 amino acid truncation at the C-terminus (Fig. S2), had no impact on NF-κB activation and did not block p65 nuclear translocation. Further screening of three clinical isolates of *S. flexneri* 2a revealed that all strains encoded a truncated OspZ protein. Similar to the truncated form of OspZ from *S. flexneri* 2a, a GFP-NleE truncation lacking the C-terminal 36 amino acid residues, GFP-NleE_1-188_ was unable to prevent NF-κB activation. However, the C-terminal region was not sufficient for this antagonism, as GFP-NleE_188-224_ did not inhibit NF-κB activation (Fig. 5C). Interestingly, in contrast to the other full length GFP-NleE/OspZ fusion proteins, GFP-OspZ from *S. flexneri* 6 and *S. flexneri* 2a was largely excluded from the nucleus (Fig. 5B). Although the molecular basis of this is unknown, it may indicate that the mechanism of action of NleE/OspZ is in the cytoplasm of the cell since GFP-OspZ_SF6_ inhibited NF-κB activation to the same degree as GFP-NleE (Fig. 5A).

### A C-terminal IDSY(M/I)K motif in NleE and OspZ is important for the inhibition of NF-κB activation

**Figure 6:**
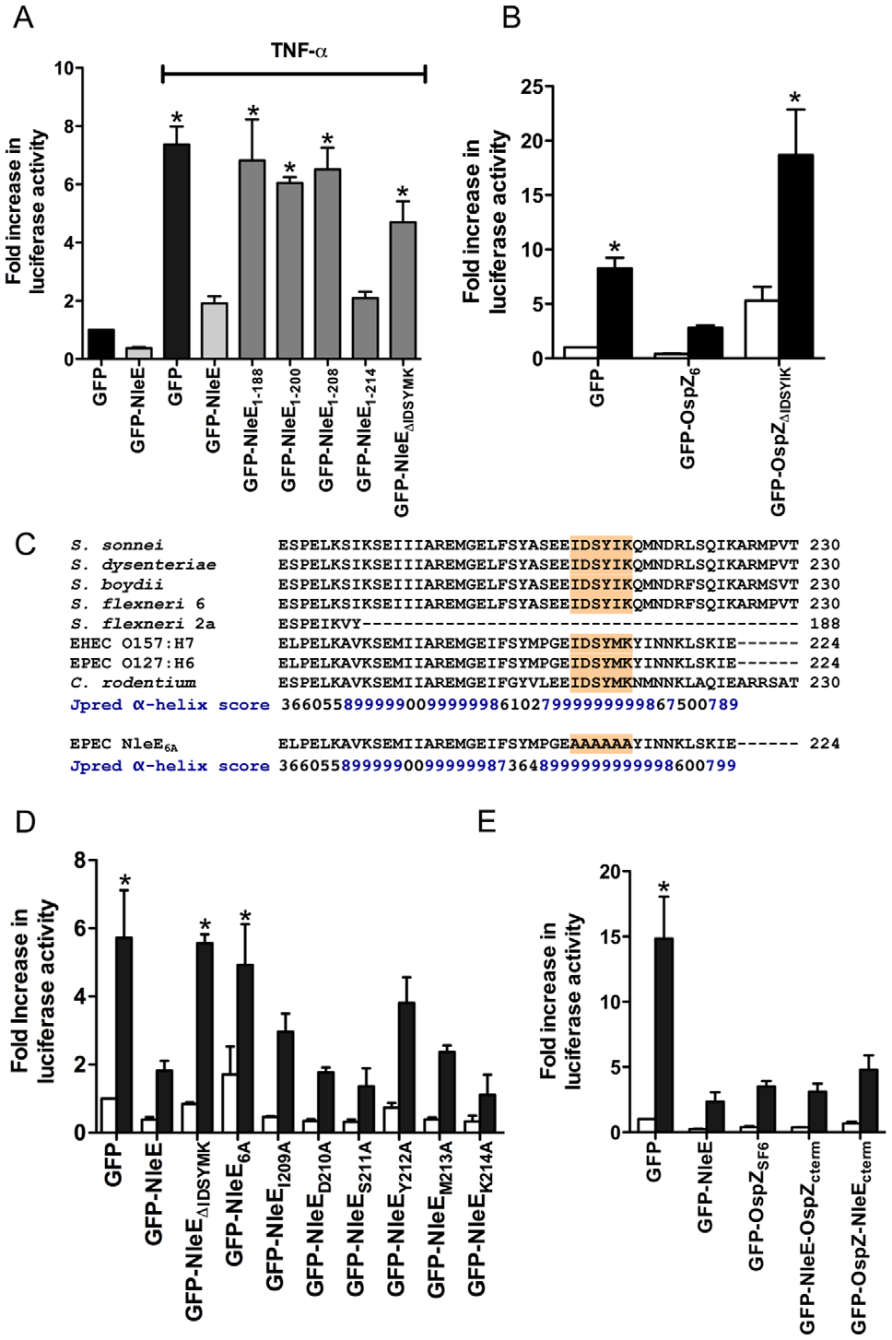
Effect of NleE deletions and mutations on NF-κB activation. **A**. Fold increase in NF-κB dependent luciferase activity in cells transfected with pEGFP-C2 or derivatives of pGFP-NleE as labelled and left unstimulated or stimulated with TNFα for 30 min where indicated. Results are the mean ± SEM of at least 3 independent experiments performed in triplicate. *significantly different to GFP-NleE stimulated with TNFα (*P*<0.05, one way ANOVA) **B**. Fold increase in NF-κB dependent luciferase activity in cells transfected with pEGFP-C2, pGFP-OspZ_6_ or pGFP-OspZ_ΔIDSYMK_ and left unstimulated (white bars) or stimulated with TNFα for 30 min (black bars). Results are the mean ± SEM of at least 3 independent experiments performed in triplicate. *significantly different to GFP-OspZ stimulated with TNFα (*P*<0.05, one way ANOVA) **C**. Alignment of the C-terminal region of NleE and OspZ from A/E pathogens and *Shigella* as well as the NleE_6A_ variant. The IDSY(M/I)K motif is shaded and the Jpred prediction for an amino acid contributing to secondary structure is given as an accuracy score between 0 and 9, where 9 represents the most reliable prediction [16]. **D**. Fold increase in NF-κB dependent luciferase activity in cells transfected with pEGFP-C2 or derivatives of pGFP-NleE as labelled and left unstimulated (white bars) or stimulated with TNFα for 30 min (black bars). Results are the mean ± SEM at least 3 independent experiments performed in triplicate. *significantly different to GFP-NleE stimulated with TNFα (*P*<0.05, one way ANOVA). **E**. Fold increase in NF-κB dependent luciferase activity in cells transfected with pEGFP-C2 or derivatives of pGFP-NleE and pGFP-OspZ as labelled and left unstimulated (white bars) or stimulated with TNFα for 30 min (black bars). Results are the mean ± SEM at least 3 independent experiments performed in triplicate. *significantly different to GFP-NleE stimulated with TNFα (*P*<0.05, one way ANOVA).

To examine further the C-terminal region of NleE and OspZ, we performed a deletion analysis of NleE. Whereas truncated NleE_1-214_, inhibited NF-κB activation to the same degree as full length NleE, truncated NleE_1-208_ was inactive (Fig. 6A). This suggested that the region between amino acid residues 208 and 214, with the motif IDSYMK was critical for NleE function. We then constructed a deleted form of NleE lacking amino acids I^209^DSYMK^214^ which was unable to inhibit NF-κB activation (Fig. 6A) as was a corresponding deleted form of OspZ from *S. flexneri* 6 lacking amino acids I^209^DSYIK^214^ (Fig. 6B). In fact GFP-OspZ_ΔIDSYMK_ appeared to have a pro-inflammatory effect even in unstimulated cells (Fig. 6B).

The C-terminal 50 amino acids of NleE and OspZ are strongly predicted by Jpred [16] to form an alpha-helical region, therefore it was possible that the IDSY(M/I)K deletion had disrupted protein secondary structure (Fig. 6C). To account for this possibility and preserve the predicted alpha helix, we changed all six amino acids to alanine to generate GFP-NleE_6A_. NleE_6A_ had the same alpha-helical prediction by Jpred as NleE (Fig. 6C). Similar to GFP-NleE_ΔIDSYMK_, GFP-NleE_6A_ was unable to inhibit NF-κB activation (Fig. 6D), and NleE_6A_ delivered by the T3SS during infection was also unable to inhibit p65 nuclear translocation (Fig. 1B). Further mutation of individual amino acids within the IDSYMK motif of NleE to alanine did not make a significant difference to NF-κB inhibition compared to the fulllength GFP-NleE fusion (Fig. 6D). Expression of all GFP-NleE derivatives was tested by immunoblot using anti-GFP antibodies to ensure that differences in activity were not due to differences in the levels of GFP fusion proteins (Fig. S3).

To determine if variations in amino acid sequence at the C-termini of NleE and full length OspZ had any functional significance (Fig. 6C), we performed a domain swap by exchanging the last 40 amino acids of NleE with the last 46 amino acids of OspZ and vice versa. Both chimeric forms of NleE and OspZ (NleE-OspZ_cterm_ and OspZ-NleE_cterm_) were fully functional (Fig. 6E) and they retained their native localization (data not shown). Therefore these regions were functionally interchangeable, suggesting that NleE and OspZ use the same molecular mechanism to inhibit NF-κB activation.

### NleB inhibits NF-κB activation through a distinct mechanism

**Figure 7:**
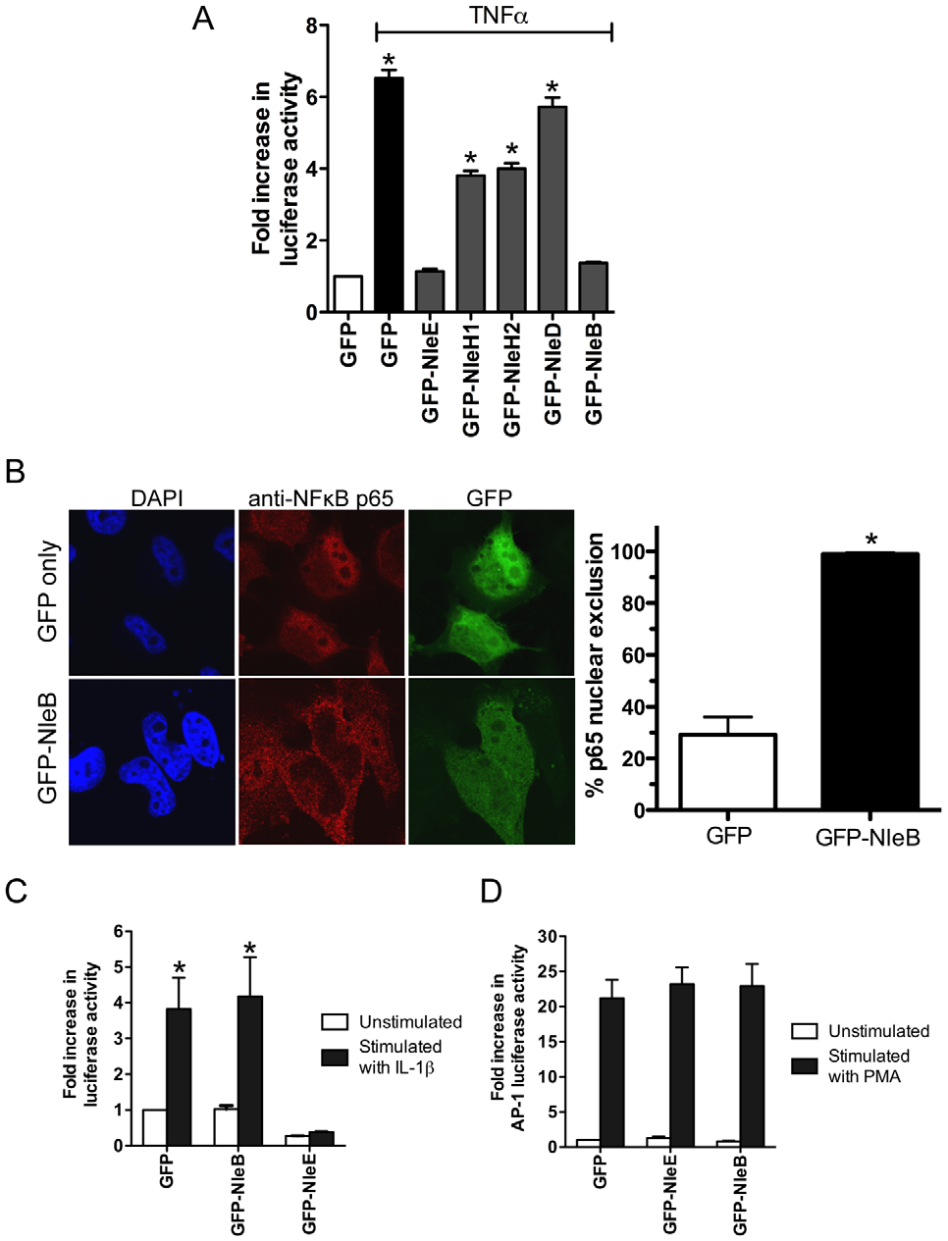
Inhibition of NF-κB activation by NleB. **A**. Fold increase in NF-κB dependent luciferase activity in HeLa cells transfected with pEGFP-C2 (GFP only) or pGFP-NleE, pGFP-NleH1, pGFP-NleH2, pGFP-NleD or pGFP-NleB and stimulated with TNFα. Results are the mean ± SEM at least 3 independent experiments performed in triplicate. *significantly different to GFP-NleE stimulated with TNFα (*P*<0.05, one way ANOVA) **B**. Representative immunofluorescence fields and quantification of nuclear exclusion using antibodies to p65 (red) of HeLa cells transfected with pGFP-C2 or pGFP-NleB (green) and stimulated with TNFα for 30 min. Results are expressed as the percentage of GFP-positive cells that exclude p65 and are the mean ± SEM of three independent experiments performed in duplicate. At least 100 GFP-positive cells were counted per test. *significantly different to GFP only stimulated with TNFα *P*<0.0005 (unpaired, two-tailed *t*-test). **C**. Fold increase in NF-κB dependent luciferase activity in HeLa cells transfected with pEGFP-C2 (GFP), pGFP-NleE or pGFP-NleB and stimulated with IL-1β. Results are the mean ± SEM at least 3 independent experiments performed in triplicate. *significantly different to GFP-NleE stimulated with IL-1β (*P*<0.05, one wayANOVA) **D**. Fold increase in AP-1 dependent luciferase activity in HeLa cells transfected with pEGFP-C2 (GFP only), pGFP-NleE or pGFP-NleB and stimulated with PMA. Results are the mean ± SEM at least 3 independent experiments performed in triplicate. There was no significant difference between any tests (*P*>0.05, one way ANOVA).

To compare the activity of NleE with another T3SS effector, NleH, reported to interfere with NF- κB activation [17], we generated GFP-NleH1 and GFP-NleH2 fusions from EPEC E2348/69 and tested these for their ability to inhibit NF-κB activity following stimulation with TNFα. In this system, NleE showed greater inhibition of NF-κB activation than either NleH1 or NleH2 (Fig. 7A). To test for possible non-specific effects on NF-κB activation of effector over-expression by transfection, we also tested the effect of NleD and NleB on NF-κB activation following stimulation with TNFα. While NleD had no effect on luciferase induction in response to TNFα, NleB inhibited NF-κB activation as effectively as NleE (Fig. 7A). NleB is encoded directly upstream from NleE and this organization is highly conserved among A/E pathogens [18]. Fluorescence microscopy of GFP-NleB transfected cells stimulated with TNFα confirmed that GFP-NleB inhibited p65 translocation (Fig. 7B).

NleE inhibited NF-κB activation in response to both TNFα and IL-1β so we tested the ability of NleB to inhibit IL-1β signaling. Whereas, GFP-NleE was effective against both TNFα and IL-1β stimulation, GFP-NleB had no effect on NF-κB activation stimulated by IL-1β (Fig. 7C). This suggested that the two effectors act at different points in the NF-κB signaling cascade. To examine the effect of NleE and NleB on other signaling pathways, we used an AP-1 reporter to monitor JNK/MAPK signaling. Neither NleE nor NleB inhibited AP-1 activation by phorbol 12-myristate 13-acetate (PMA) (Fig. 7D), and NleB also had no effect on STAT1/2 activation (data not shown). This suggests that the effectors target only a subset of signaling pathways involving NF-κB.

**Figure 8:**
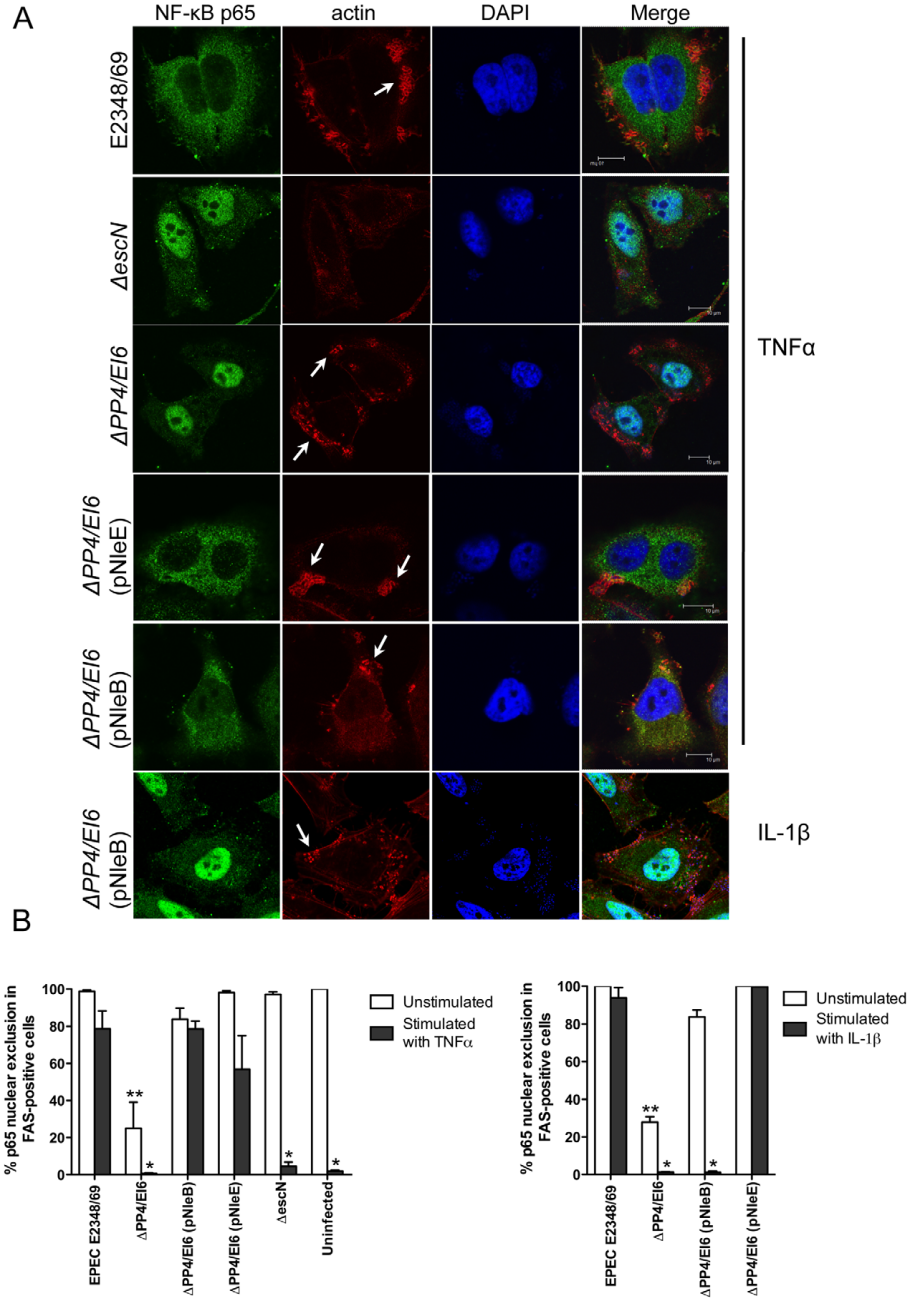
Effect of bacterially injected NleB and NleE on NF-κB activation. **A**. Representative immunofluorescence fields showing p65 staining (green) in FAS-positive HeLa cells (red) infected with derivatives of EPEC E2348/69, stimulated with TNFα or IL-1β as indicated and stained for nucleic acid with DAPI (blue). Arrows indicate FAS-positive lesions. **B**. Quantification of p65 nuclear exclusion in cells infected with derivatives of EPEC E2348/69 and stimulated with TNFα or IL-1β as indicated. Results are expressed as the percentage of FAS-positive cells that exclude p65 from the nucleus and are the mean ± SEM of three independent experiments performed in duplicate. At least 100 FAS-positive cells were counted per test. *significantly different to E2348/69 stimulated with TNFα or IL-1β **significantly different to E2348/69 left unstimulated (*P*<0.05, one way ANOVA).

To ensure that NleE and NleB translocated by the LEE-encoded T3SS conferred the same phenotype as ectopic expression of the effectors by transfection, we infected HeLa cells with wild type EPEC E2348/69 and a double island mutant that lacked the genomic regions, PP4 and IE6 [19]. The double island mutant was used to eliminate genes encoding NleE and NleB in IE6 as well as NleB2, a close homologue of NleB, encoded in PP4 [19]. The ΔPP4/IE6 island mutant was complemented with pNleE or pNleB to examine the contribution of each effector to the inhibition of p65 translocation. In unstimulated cells, there was no difference in p65 nuclear translocation between uninfected cells and those infected with wild type EPEC E2348/69 or the T3SS mutants (*escN* and *escF*) (Fig. 8A and B and Fig. 1B). This suggested that, over a 4 h infection, bacterial products such as flagellin and lipopolysaccharide were not sufficient to stimulate signaling. In contrast, the ΔPP4/IE6 island mutant induced substantial p65 nuclear translocation (Fig. 8B), which may indicate that the translocation and/or biochemical function of some effectors is proinflammatory. In infected cells, both NleB and NleE injected by the T3SS had the capacity to inhibit p65 nuclear translocation in response to TNFα but only NleE was effective in response to IL-1β (Fig. 8A and B).

### Effect of NleE, NleB and OspZ on IκB degradation

**Figure 9:**
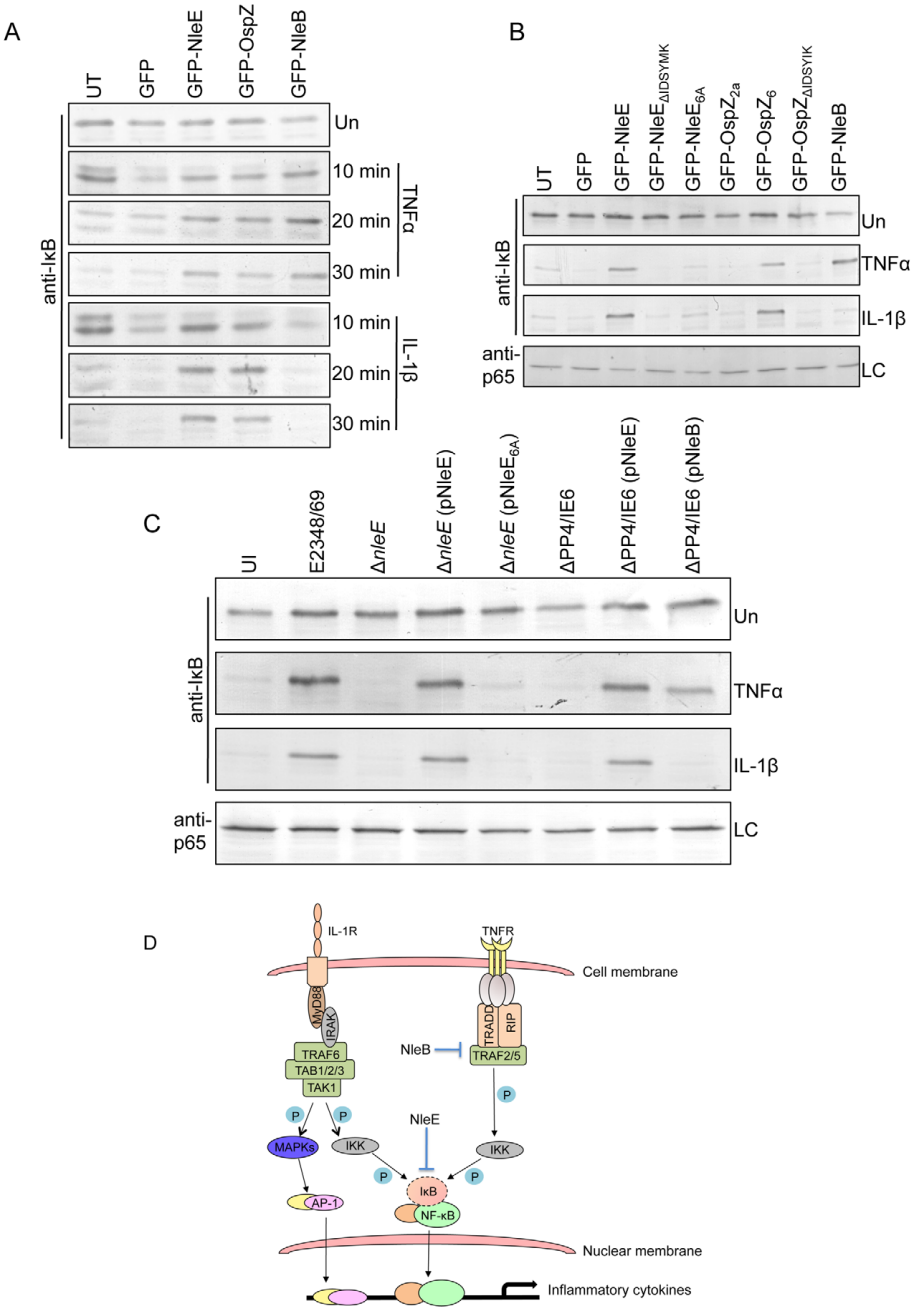
Effect of NleB and NleE on IκB degradation. **A**. Time course of IκB degradation in HeLa cells transfected with pEGFP-C2, pGFP-NleE, pGFP-OspZ and pGFP-NleB. Cells were stimulated with TNFα or IL-1β for the times indicated and IκB was detected by immunoblot using anti-IκB antibodies. UT, untransfected. Un, unstimulated **B**. IκB degradation in HeLa cells transfected with GFP-NleE and GFP-OspZ variants and GFP-NleB and stimulated with TNFα or IL-1β for 30 min. IκB was detected by immunoblot using anti-IκB antibodies. p65 was used as a loading control (LC) and detected using anti-p65 antibodies. A representative immunoblot is shown. UT, untransfected. Un, unstimulated **C**. Infection of HeLa cells with derivatives of EPEC E2348/69 for 90 min followed by stimulation with TNFα or IL-1β for 30 min. Cells were collected for immunoblotting with anti-IκB antibodies. p65 was used as a loading control (LC) and detected using anti-p65 antibodies. A representative immunoblot is shown. UI, unininfected. Un, unstimulated **D**. Proposed model of the inhibition of NF-κB signaling by EPEC. Components of both the TNFα and IL-1β pathways are labelled and the predicted points at which NleE and NleB act on the pathways are shown as blocked arrows. The dashed line represents IκB degradation and shaded (P) represents phosphorylation.

Since EPEC infection has been reported to inhibit IκB degradation [12], a critical event in the activation of NF-κB, the effect of NleE and NleB on IκB degradation was examined here in TNFα and IL-1β stimulated cells. Ectopically expressed GFP-NleE and GFP-OspZ inhibited IκB degradation in response to both stimuli whereas GFP-NleB inhibited IκB degradation in response to TNFα only (Fig. 9A). GFP-NleE_6A_ lacked the ability to inhibit IκB degradation as did GFP-NleE_ΔIDSYMK_ and GFP-OspZ_ΔIDSYMK_ (Fig. 9B). In addition, we tested whether NleB and NleE delivered by the T3SS had the same effect as ectopically expressed protein on IκB degradation stimulated by TNFα and IL-1β. Wild type EPEC E2348/69, Δ*nleE* (pNleE), ΔPP4/IE6 (pNleE) or ΔPP4/IE6 (pNleB) inhibited IκB degradation in HeLa cells stimulated with TNFα but IκB degradation was not inhibited in cells infected with ΔPP4/IE6 (pNleB) and stimulated with IL-1β. This suggests that NleE and NleB act in different ways to interfere with NF-κB signaling (Fig. 9C).

## Discussion

The activation of NF-κB signaling is a critical host response to infection. In this study, we found that the T3SS effector NleE from EPEC prevented nuclear translocation of the p65 NF-κB subunit, leading to diminished *IL8* expression and a compromised IL-8 response. The inhibition of p65 nuclear translocation occurred when NleE was expressed ectopically or when NleE was delivered through the T3SS by infection. We also observed that NleE inhibited nuclear translocation of c-Rel but not nuclear import of activated p50, STAT1 or STAT2. Both p65 and c-Rel are structurally similar and contain transcriptional activation domains that initiate gene expression [6]. In contrast, p50 lacks a transcriptional activation domain, so that p50/p50 homodimers act as transcriptional repressors. Thus, NleE appears to obstruct nuclear translocation of Rel family transcriptional activators while allowing nuclear import of a transcriptional repressor, resulting in the suppression of *IL8* expression. The selectivity of NleE for p65 and c-Rel is not unprecedented as lack of nuclear translocation of p65 and c-Rel but not p50 was recently reported for oestrogen-induced inhibition of NF-κB activation, although the mechanism is unknown [20].

NleE is one of the conserved core type III effectors of A/E pathogens [19]. We observed that ectopically expressed NleE from EHEC O157:H7 and the murine pathogen, *C. rodentium* also inhibited NF-κB activation and p65 translocation. A close homologue of NleE, OspZ, is found in *Shigella*, however in *S. flexneri* 2a, OspZ is truncated to the length of NleE_1-188_ [10]. Both the truncated form of OspZ from *S. flexneri* 2a and a C-terminal 36 amino acid deletion mutant of NleE were inactive, suggesting that the C-terminus was critical for the immunosuppressive function of NleE. However, this region was not sufficient for inhibition of p65 nuclear translocation as a region encompassing the last 36 amino acids of NleE alone was unable to prevent NF-κB activation. A domain swap between NleE and OspZ of the last ∼40 amino acids showed that these regions were functionally interchangeable and we identified a 6-amino acid motif, IDSY(M/I)K, that was critical for both NleE and OspZ function.

Although A/E pathogens stimulate an inflammatory response *in vivo* and proteins such as flagellin are recognised by TLR5 [21], [22], previous work has suggested that A/E pathogens modulate that inflammatory response by inhibiting p65 nuclear translocation as well as IκB degradation [11], [12], [23]. Here, we found that NleE inhibited nuclear translocation of p65 by preventing IκB degradation in response to TNFα and IL-1β. In contrast, we found that NleB inhibited IκB degradation in response to TNFα only. Since TNFα and IL-1β signaling converges at the point of IKK phosphorylation (Fig. 9D) [24], NleE may act on IKK or IκB itself to prevent IκB degradation. TAK1 or other MAPK may also have involvement in IKK phosphorylation leading to JNK activation [24], however, JNK signaling, represented here by the AP-1 reporter, was not affected by NleE and so we predict that NleE interferes with IKK or IκB function directly. Indeed while this work was under review, Nadler *et al* reported that NleE inhibits IKK phosphorylation [25]. The authors also proposed that NleB assists the inhibition of IκB degradation by NleE [25]. Here we hypothesize that NleB acts upstream of IKK in the TNFα pathway since NleB did not inhibit IκB degradation in response to IL-1β (Fig. 9D). We therefore propose a model where NleE and NleB act at different points in the NF-κB signaling pathway and each plays a distinct role in the inhibition of p65 nuclear translocation. In the TNFα pathway, NleE and NleB have overlapping and somewhat redundant inhibitory roles as complementation of the ΔPP4/IE6 double island mutant with either NleE or NleB was sufficient to block p65 nuclear translocation. In the IL-1β pathway however, NleB was not able to compensate for the lack of NleE. Although we believe that NleB acts independently of NleE, these results do not exclude the possibility that in IL-1β stimulated cells, NleB acts in concert with NleE [25].

The fact that both NleE and NleB inhibit NF-κB activation raises the possibility that more effectors contribute to the suppression of innate signaling pathways. Although compromised compared to wild type EPEC, the *nleE* mutant showed significantly greater inhibition of IL-8 secretion than an T3SS mutant, which lacks the ability to translocate all T3SS effectors. While one of the additional effectors inhibiting p65 translocation is clearly NleB, a close homologue, NleB2 may also have anti-inflammatory activity and perhaps other effectors in the genomic islands, PP4 and IE6. In addition, NleH1 and NleH2 were recently reported to interfere with the activation of NF-κB by binding ribosomal protein S3 (RPS3), a co-factor of nuclear NF-κB complexes, and sequestering it in the cytoplasm [17]. We also found that ectopically expressed NleH1 and NleH2 inhibited NF-κB activation, but not to the same degree as NleE and NleB. Together these anti-inflammatory effectors may balance the action of other effectors that through their biochemical activity stimulate inflammatory signaling, as suggested by the ΔPP4/IE6 double island mutant, which showed increased p65 nuclear translocation in uninfected cells compared to a T3SS mutant. Therefore, despite the fact that EPEC and *Shigella* infection ultimately induces gut inflammation, we propose that NleE/OspZ and NleB contribute to pathogenesis by inhibiting an initial host inflammatory response to allow the bacteria to persist in the early stages of infection.

A multi-effector attack on NF-κB signaling occurs during *Shigella* infection, which modulates NF-κB activation through the effectors OspG and OspF [3], [26]. Our studies suggest that *Shigella* strains carrying full length OspZ have evolved a further distinct mechanism to modulate NF-κB signaling. This makes the absence of a functional OspZ protein in *S. flexneri* 2a curious and may also explain previous findings that OspZ from *S. flexneri* 2a potentially enhanced inflammation by inducing polymorphonucleocyte migration across a polarized epithelium [10]. The truncation rendering OspZ inactive in *S. flexneri* was serotype specific however, as *S. flexneri* 6 encoded functional full length OspZ, similar to *S. boydii*.

In this study, we have ascribed a function to the NleE/OspZ family of T3SS effectors shared by attaching and effacing pathogens and *Shigella* as well as the EPEC effector, NleB. Despite the remarkably different infection strategies of these two groups of pathogens, they appear to have a mutual need to inhibit the host inflammatory response during infection. NleE, NleB and OspZ are the latest T3SS effectors to target NF-κB activation and the expression of NF-κB-dependent genes. Neither NleE nor NleB inhibited STAT1/2 or AP-1 signaling, suggesting that the proteins target the NF-κB pathway specifically. The ongoing identification of T3SS effectors that act on this and other inflammatory pathways will continue to provide insight into the molecular mechanisms by which bacterial pathogens inhibit immune signaling and establish infection.

## Methods

### HeLa and Caco-2 infections, IL-8 secretion and expression

The bacterial strains and plasmids used in this study are listed in Table 1. The construction of vectors and culturing of bacterial strains for infection is described in detail in the supplementary methods (Protocol S1). EPEC strains were used to infect HeLa cells for 4 h without exogenous stimulation or for 90 min after which the media was replaced with DMEM supplemented with 20 ng/ml TNFα or 10 ng/ml IL-1β (eBioscience, San Diego, CA) and the infection was continued for a further 30 min. For Caco-2 cells, EPEC infection continued for 4 h after which the cells were washed, treated with 100 µg/ml gentamicin for 2 h. For mRNA analysis monolayers were incubated for 3 h in media supplemented with 50 ug/ml gentamicin with or without 5 ng/ml IL-1β. For analysis of IL-8 secretion, monolayers were infected for 4 h and incubated for 24 h in media supplemented with 50 ug/ml gentamicin with or without 5 ng/ml IL-1β. IL-8 secretion into cell culture supernatants was measured by ELISA (Peprotech EC). The expression of *IL8* from total RNA was determined using the comparative quantification method included in Rotor-Gene 1.7 software (Qiagen) as described in the supplementary methods using gene specific primers (Protocol S1).

**Table 1:** Bacterial strains and plasmids used in this study.

Strains	Characteristics	Source/reference
EPEC E2348/69	Wild type EPEC O127:H6	[27]
Δ*nleE*	EPEC E2348/69 Δ*nleE* Cm^R^	[10]
ΔPP4/IE6	EPEC E2348/69 PP4/IE6 double island deletion	O. Marchés
EHEC EDL933	Wild type EHEC O157:H7	[28]
*C. rodentium* ICC169	Spontaneous nalidixic acid resistant derivative of wild-type *C. rodentium* biotype 4280 (Nal^R^)	[29]
*S. flexneri* 2104	Wild type *S. flexneri* serotype 2a	Roy Robins-Browne
*S. flexneri* 0106164	Wild type *S. flexneri* serotype 6	Roy Robins-Browne
*S. boydii* SBA1384	Wild type *S. boydii* serotype 4	Ben Adler
**Plasmids**		
pTrc99A	Cloning vector for expression of proteins from P*trc*	Pharmacia Biotech
pNleE	*nleE* from EPEC E2348/69 in pTrc99A	This study
pNleE_6A_	*nleE* from EPEC E2348/69 in pTrc99A carrying alanine substitutions for each amino acid in the IDSYMK motif	This study
pNleB	*nleB* from EPEC E2348/69 in pTrc99A	This study
pEGFP-C2	Green fluorescent protein (GFP) expression vector	Clontech
pGFP-NleE	Full length *nleE* from EPEC E2348/69 in pEGFP-C2	[10]
pGFP-NleE_O157_	Full length *nleE* from EHEC EDL933 in pEGFP-C2	This study
pGFP-NleE_CR_	Full length *nleE* from *C. rodentium* ICC169 in pEGFP-C2	This study
pGFP-OspZ_SF2a_	Full length *ospZ* from *S. flexneri* 2104 in pEGFP-C2	This study
pGFP-OspZ_SF6_	Full length *ospZ* from *S. flexneri* 0106164 in pEGFP-C2	This study
pGFP-OspZ_SB_	Full length *ospZ* from *S. boydii* SBA1384 in pEGFP-C2	This study
pGFP-NleH1	Full length *nleH1* from EPEC E2348/69 in pEGFP-C2	This study
pGFP-NleH2	Full length *nleH2* from EPEC E2348/69 in pEGFP-C2	This study
pGFP-NleD	Full length *nleD* from EPEC E2348/69 in pEGFP-C2	This study
pGFP-NleB	Full length *nleB* from EPEC E2348/69 in pEGFP-C2	This study
pGFP-NleE_1-188_	Truncation of *nleE* from EPEC E2348/69 encoding amino acids 1-188 in pEGFP-C2	This study
pGFP-NleE_1-200_	Truncation of *nleE* from EPEC E2348/69 encoding amino acids 1-200 in pEGFP-C2	This study
pGFP-NleE_1-208_	Truncation of *nleE* from EPEC E2348/69 encoding amino acids 1-208 in pEGFP-C2	This study
pGFP-NleE_1-214_	Truncation of *nleE* from EPEC E2348/69 encoding amino acids 1-214 in pEGFP-C2	This study
pGFP-NleE_ΔIDSYMK_	Full length *nleE* from EPEC E2348/69 carrying the IDSYMK deletion in pEGFP-C2	This study
pGFP-NleE_6A_	Full length *nleE* from EPEC E2348/69 in pEGFP-C2 carrying alanine substitutions for each amino acid in the IDSYMK motif	This study
pGFP-NleE_I209A_	Full length *nleE* from EPEC E2348/69 carrying the mutation I209A in pEGFP-C2	This study
pGFP-NleE_D210A_	Full length *nleE* from EPEC E2348/69 carrying the mutation D210A in pEGFP-C2	This study
pGFP-NleE_S211A_	Full length *nleE* from EPEC E2348/69 carrying the mutation S211A in pEGFP-C2	This study
pGFP-NleE_Y212A_	Full length *nleE* from EPEC E2348/69 carrying the mutation Y212A in pEGFP-C2	This study
pGFP-NleE_M213A_	Full length *nleE* from EPEC E2348/69 carrying the mutation M213A in pEGFP-C2	This study
pGFP-NleE_K214A_	Full length *nleE* from EPEC E2348/69 carrying the mutation K214A in pEGFP-C2	This study
pGFP-OspZ_ΔIDSYIK_	Full length *ospZ* from *S. flexneri* carrying the IDSYIK deletion in pEGFP-C2	This study
pGFP-NleE-OspZ_cterm_	NleE_1-183_ fused to the last 46 amino acids of OspZ	This study
pGFP-OspZ-NleE_cterm_	OspZ_1-183_ fused to the last 40 amino acids of NleE	This study
pRL-TK	Renilla luciferase vector	Promega
pNF-κB-Luc	Vector for measuring NF-κB dependent luciferase expression	Clontech
p(9-27)4th(–39)Lucter	Vector for measuring STAT1/2 dependent luciferase expression	[15]
pAP-1-Luc	Vector for measuring AP-1 dependent luciferase expression	Clontech

### Immunofluorescence, fluorescence actin staining test and confocal microscopy

Plasmids were transfected into HeLa cells for ectopic expression of GFP fusion proteins using Lipofectamine 2000 in accordance with the manufacturer's recommendation (Invitrogen, Carlsbad CA, USA). Transfected HeLa cells were treated with 20 ng/ml TNFα, 10 ng/ml IL-1β or IFNα (500 U/ml; Calbiochem, La Jolla, CA,USA) for 30 min at 37°C and 5% CO_2_. Transfected or infected cells were fixed in 3.7% (wt/vol) formaldehyde (Sigma) in PBS for 10 min and permeablized with acetone-methanol (1∶1, vol/vol) at −20°C for 15 min. Following a 30 min blocking in PBS with 3% (wt/vol) bovine serum albumin (Amresco, Ohio, USA) samples were exposed to rabbit polyclonal anti-p65 (SC-109, Santa Cruz, Santa Cruz CA, USA), anti-c-Rel (#4727, Cell Signaling, Beverly MA, USA), anti-STAT1 (SC-345, Santa Cruz), anti-STAT2 (SC-476, Santa Cruz) or mouse monoclonal anti-p50 (2E6, Novus Biologicals, Littleton CO, USA). Antibodies were used at a 1∶100, or 1∶50 for anti-c-Rel, (vol/vol) diluted in blocking solution for 1 h at 20°C. Alexa Fluor 488 or Alexa Fluor 568 (Invitrogen) conjugated anti-mouse or anti-rabbit immunoglobulin G were used at 1∶2000. Coverslips were mounted onto microscope slides with Prolong Gold containing 4′,6-diamidino-2-phenylindole (DAPI; Invitrogen). For the fluorescence actin staining (FAS) test, HeLa and Caco-2 cells were infected with bacterial strains, fixed and permeabilized as described above and cells were incubated with 0.5 mg/ml phalloidin conjugated to rhodamine for 30 min. Images were acquired using a confocal laser scanning microscope (Leica LCS SP2 confocal imaging system) with a 100x/1.4 NA HCX PL APO CS oil immersion objective. Nuclear exclusion of NF-κB, STAT1 and STAT2 was quantified from at least 3 independent experiments for both transfection and infection studies.

### NF-κB, STAT and AP-1 reporter assay

To examine the activity of NF-κB, a dual luciferase reporter system was employed. HeLa cells were seeded into 24-well trays and co-transfected with derivatives of pEGFP-C2 (1.0 µg) together with 0.2 µg of pNF-κB-Luc (Clontech, Palo Alto CA, USA) and 0.04 µg of pRL-TK (Promega, Madison WI, USA). Approximately 24 h after transfection, cells were left untreated or stimulated with 20 ng/ml TNFα or 10 ng/ml IL-1β for 16 h. Firefly and *Renilla* luciferase levels were measured using the Dual-luciferase reporter assay system (Promega) in the Topcount NXT instrument. For each sample, the expression of firefly luciferase was normalized for *Renilla* luciferase measurements and NF-κB activity was expressed relative to unstimulated pEGFP-C2 transfected cells.

To measure the induction of STAT1 and 2, the IFN-α/β-responsive luciferase reporter plasmid p(9-27)4th(–39)Lucter (ISRE-Luc) [15] was used in combination with the Renilla luciferase plasmid pRL-TK. HeLa cells were transfected with both plasmids as described above and stimulated with IFNα (500 U/ml; Calbiochem) for 30 min. Luciferase activity was measured as described above. To measure the induction of AP-1, the cAMP response element (CRE)-dependent luciferase vector pAP-1-Luc was used in combination with the Renilla luciferase plasmid pRL-TK. HeLa cells were transfected with both plasmids as described above and stimulated with 25 ng/ml phorbol 12-myristate 13-acetate (PMA) for 30 min. Luciferase activity was measured as described above.

### Detection of IκB degradation by immunoblot

To test the effect of ectopically expressed NleE, NleB and OspZ and on IκB degradation, HeLa cells were mock transfected or transfected with pEGFP-C2 or pEGFP-NleE, pEGFP-NleB, pEGFP-OspZ and derivatives and incubated for 16 h before being left untreated or treated with TNF-α or IL-1β for 10, 20 or 30 min. Cell lysis was performed by incubating cells in cold lysis buffer (50 mM Tris-HCl pH 8.0, 150 mM NaCl, 5 mM EDTA, 1% NP-40) on ice for 5 min before collecting lysate and incubating on ice for a further 10 min. Cell debri was pelleted and equal volumes of supernatant were collected for SDS-PAGE. Proteins transferred to nitrocellulose membranes were probed with mouse monoclonal anti-IκBα (Cell signaling) diluted 1∶1000 or rabbit polyclonal anti-p65 (Santa Cruz) diluted 1∶1000. For infection studies, HeLa cells were infected with derivatives of EPEC E2348/69 for 90 min before stimulation with TNF-α or IL-1β for 30 min as described above.

## Supporting Information

Figure S1 CaCo-2 cells infected with derivatives of EPEC E2348/69, stimulated with IL-1β where indicated and stained for actin (green), nucleic acid (blue) and p65 (red).
(2.10 MB TIF)

Figure S2 Alignment of NleE and OspZ from A/E pathogens and *Shigella* species showing amino acid conservation.
(0.92 MB TIF)

Figure S3 Ectopic expression of GFP-NleE and GFP-OspZ derivatives in HeLa cells.
(0.73 MB TIF)

Protocol S1 Supplementary methods.
(0.08 MB DOC)
