# Supplementary figures and images for: The Type III Effectors NleE and NleB from Enteropathogenic E. coli and OspZ from Shigella Block Nuclear Translocation of NF-κB p65
Source: PLoS Pathog. 2010 May 13;6(5):e1000898. doi: 10.1371/journal.ppat.1000898 (PMC2869321; doi:10.1371/journal.ppat.1000898)

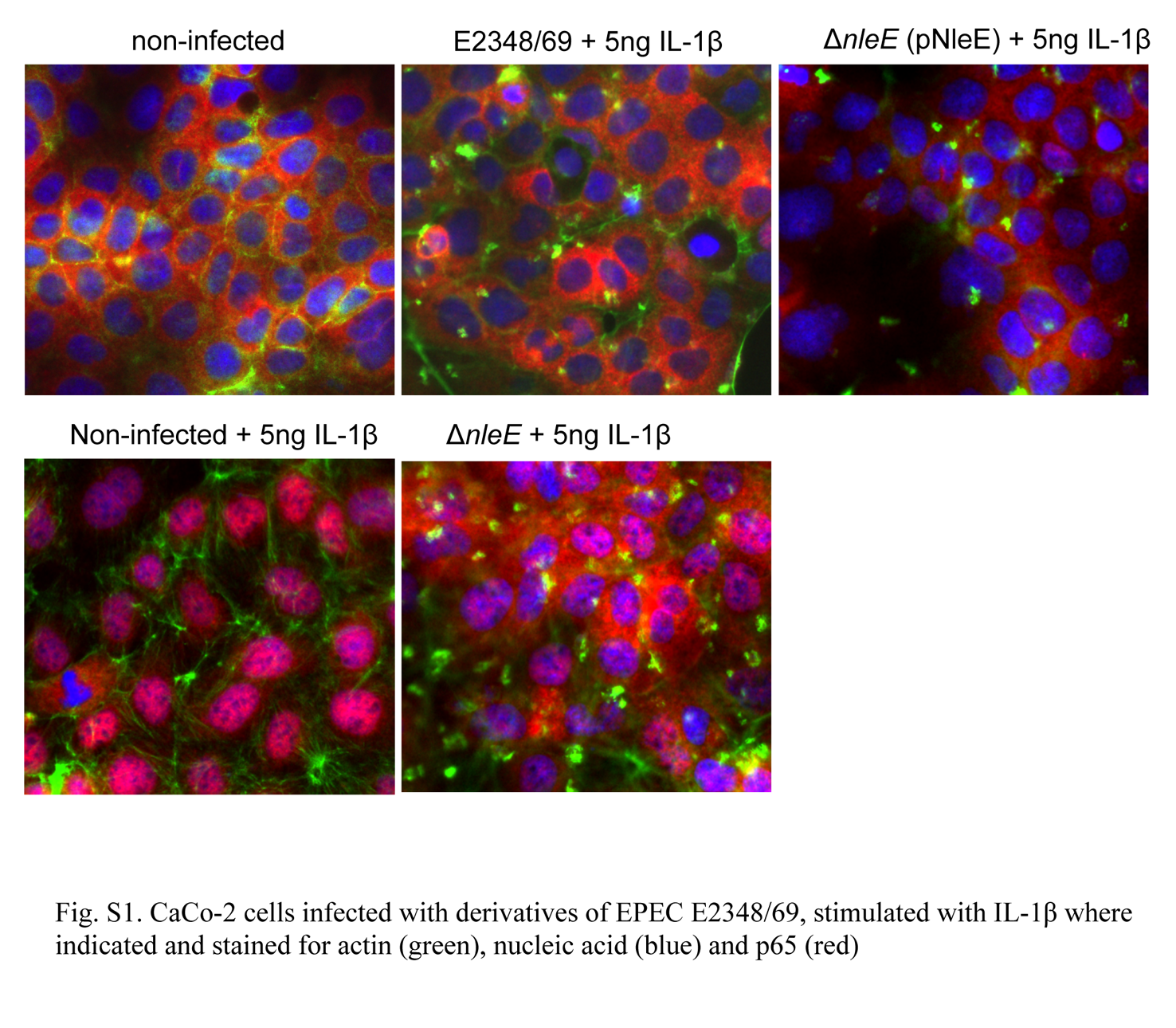

Supplement: Figure S1 — CaCo-2 cells infected with derivatives of EPEC E2348/69, stimulated with IL-1β where indicated and stained for actin (green), nucleic acid (blue) and p65 (red). (2.10 MB TIF) [file ppat.1000898.s001.tif]

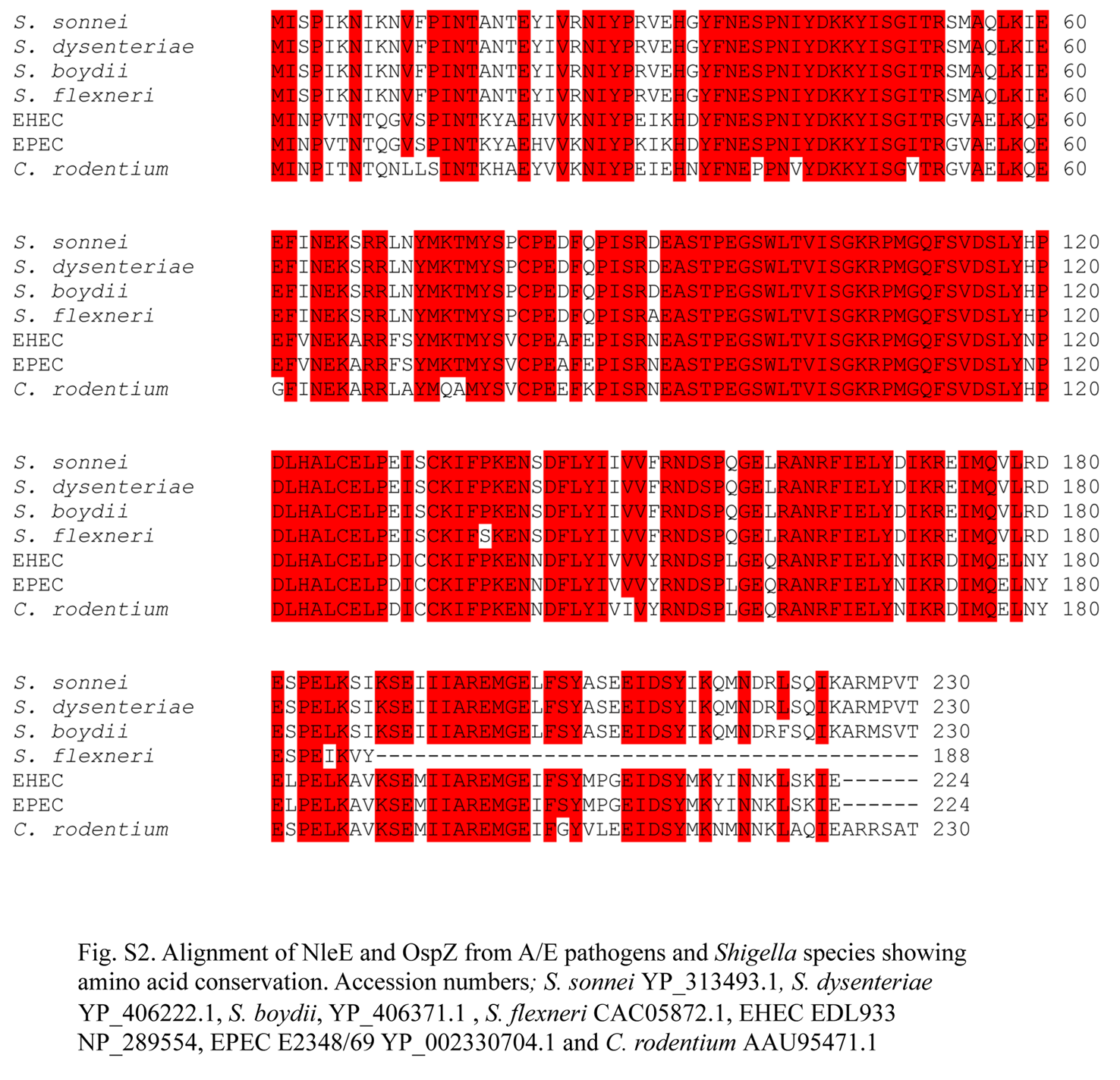

Supplement: Figure S2 — Alignment of NleE and OspZ from A/E pathogens and Shigella species showing amino acid conservation. (0.92 MB TIF) [file ppat.1000898.s002.tif]

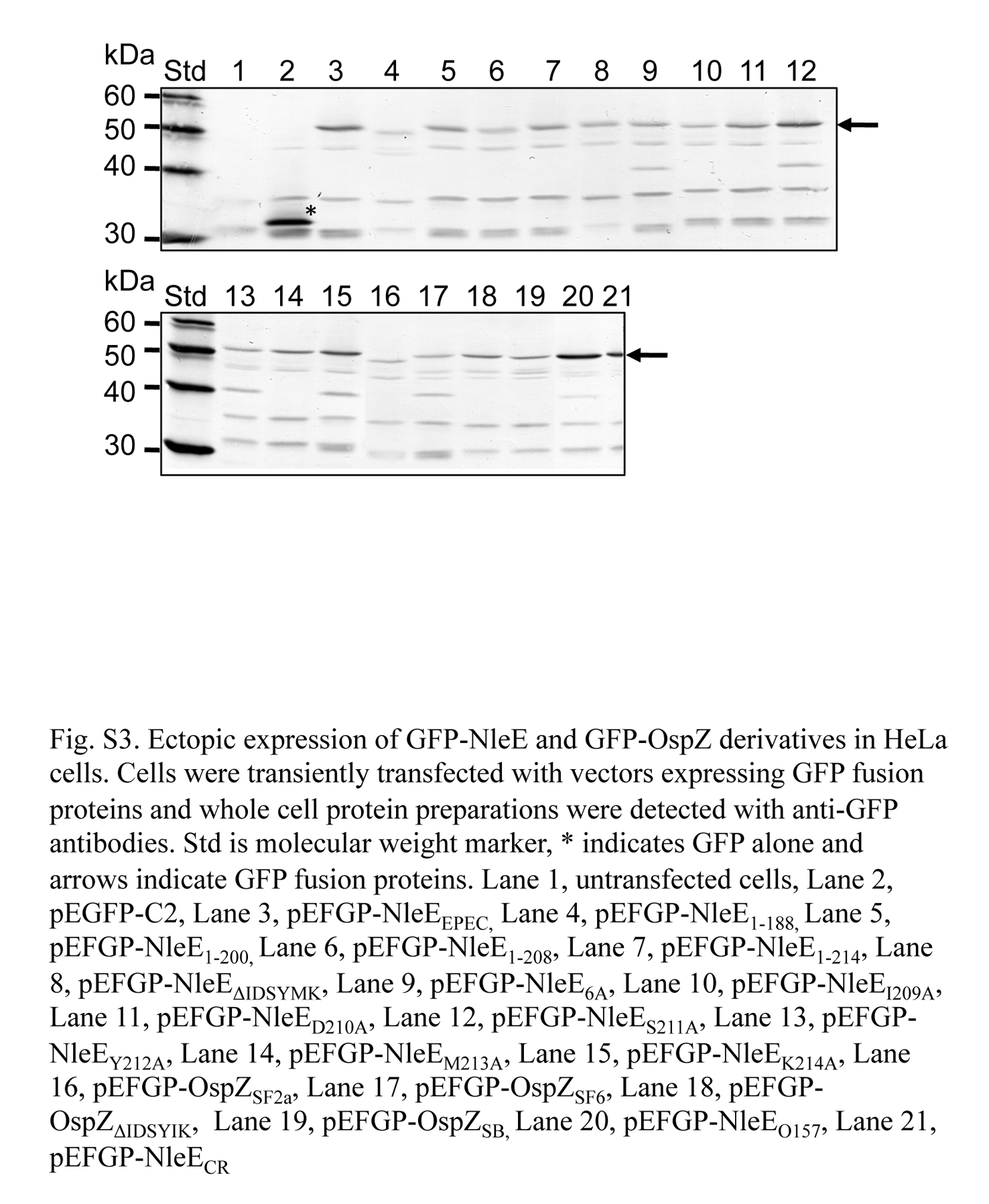

Supplement: Figure S3 — Ectopic expression of GFP-NleE and GFP-OspZ derivatives in HeLa cells. (0.73 MB TIF) [file ppat.1000898.s003.tif]
