# Supplementary material for: The Type III Effectors NleE and NleB from Enteropathogenic E. coli and OspZ from Shigella Block Nuclear Translocation of NF-κB p65
Source: PLoS Pathog. 2010 May 13;6(5):e1000898. doi: 10.1371/journal.ppat.1000898 (PMC2869321; doi:10.1371/journal.ppat.1000898)
Supplement: Protocol S1 — Supplementary methods. (0.08 MB DOC) [file ppat.1000898.s004.doc]

**SUPPLEMENTARY METHODS**

**DNA cloning, purification, and sequence analysis.** DNA-modifying enzymes were used in accordance with the manufacturer's recommendations (Promega, WI). PCR amplification consisted of an initial denaturation step at 94^o^C for 2 min, followed by 30 cycles of 44 seconds at 94^o^C, 40 seconds at 40^o^C (45^o^C for genes from *Shigella spp*.) and 1 minute at 70^o^C. PCR products and restriction digests were purified using the Perfectprep gel cleanup kit (Eppendorf, Hamburg, Germany). Full-length *nleE* gene of EPEC E2348/69 was amplified from genomic DNA by PCR using the primer pair NleE_F_/NleE_R_ (Table S1). The PCR product was digested with EcoR1/BamH1 and ligated into pEGFP-C2 to generate an N-terminal GFP fusion to NleE (pGFP-NleE). pGFP-NleB, pGFP-NleD, pGFP-NleH1 and pGFP-NleH2 were constructed using an identical strategy to pGFP-NleE using EPEC E2348/69 as a template and the primer pairs NleB_F_/NleB_R_, NleD_F_/NleD_R_, NleH1_F_/NleH2_R_ and NleH2_F_/NleH2_R_ respectively.

pGFP-NleE was further used as template for cloning. Fragments comprising 564 bp, 600 bp, 624 bp and 642 bp of *nleE* were amplified by PCR using primers NleE_F_/NleE1-188_R-C2_, NleE_F_/NleE1-200_R-C2_, NleE_F_/NleE1-208_R-C2_ and NleE_F_/NleE1-214_R-C2_, respectively (Table S1). PCR products were digested with EcoR1/BamH1 and ligated into pEGFP-C2 to generate pGFP-NleE_1-188_, pGFP-NleE_1-200_, pGFP-NleE_1-208_ and pGFP-NleE_1-214_. To obtain pGFP-NleE_188-224_ the last 108 bp of *nleE* were amplified by PCR using the pair of primers NleE188-224_F_/NleE_R_ (Table S1). After digestion with EcoR1/BamH1, the PCR product was ligated into pEGFP-C2 to generate pGFP-NleE_188-224_. Six site-directed mutants, I209A, D201A, S211A, Y2112A, M213A and K214A were obtained using the primer NleE_F_ with one of NleE_I209A-R_, NleE_D210A-R_, NleE_S211A-R_, NleE_Y212A-R_, NleE_M213A-R_, NleE_K214A-R_ and the EcoR1/BamHI fragments were fused to pEGFP-C2, generating pGFP-NleE_I209A_ pGFP-NleE_D210A_ pGFP-NleE_S211A_ pGFP-NleE_Y212A_, pGFP-NleE_M213A_, pGFP-NleE_K214A_. To create pGFP-NleE_ΔIDSYMK_ we used pGFP-NleE_1-208_ as template and the pair of primers NleE_F_/NleE_ΔIDSYMK-R_. To create pGFP-NleE_6A_, we used the primers NleE_F_ and NleE6A_R_ using pGFP-NleE as a template and the resulting PCR product was digested with EcoRI and BamHI and cloned into pEGFP-C2.

The plasmid, pGFP-NleE_O157_ was obtained by amplifying *nleE* gene from EHEC O157:H7 EDL933 using the primer pair NleE_F_/NleE_R_. The EcoR1/BamH1 fragment was then ligated into pEGFP-C2 to generate an N-terminal GFP fusion. The plasmid, pGFP-NleE_CR_, was obtained by amplifying *nleE* gene from *C. rodentium* using the primer pair NleE_CR-F_/NleE_CR-R_. The EcoR1/BamH1 fragment was then ligated into pEGFP-C2 to generate an N-terminal GFP fusion. The plasmids, pGFP-OspZ_SF_ and pGFP-OspZ_SB_ were generated by amplifying *ospZ* from *S. flexneri* and *S. boydii* using the primer pairs OspZ_F_/OspZ_SF-R_ and OspZ_F_/OspZ_SB-R_, respectively. The EcoR1/BamH1 fragments were then ligated into pEGFP-C2 to generate the corresponding N-terminal GFP fusions. To create pGFP-OspZ_ΔIDSYIK_, we generated a truncated PCR product comprising OspZ_1-208_ using the primers OspZ_F_ and OspZ208_R_ and used this as a template for a second PCR using the primers OspZ_F_ and OspZ_ΔIDSYIK-_. The resulting PCR product was digested EcoRI and BamHI and cloned into pEGFP-C2.

To create pGFP-NleE-OspZ_cterm_, we amplified NleE_1-183_ using NleE_F_ and NleE183_R_ and OspZ_184-230_ using OspZ184_F_ and OspZ_R_. The products were combined to form the template for NleE-OspZ_cterm_ using the primers NleE_F_ and OspZ_R_ and the resulting PCR product was cloned into the EcoRI and BamHI sites of pEGFP-C2. To create pGFP-OspZ-NleE_cterm_, we amplified OspZ_1-183_ using OspZ_F_ and OspZ183_R_ and NleE_184-224_ using the primers NleE184_F_ and NleE_R_. The products were combined to form the template for OspZ-NleE_cterm_ using the primers OspZ_F_ and NleE_R_ and the resulting PCR product was cloned into the EcoRI and BamHI sites of pEGFP-C2.

To generate the complementing vector, pNleE, *nleE* from pGFP-NleE was digested with EcoRI and BamHI and ligated into pTrc99A. To create pNleE_6A_, *nleE_6A_* was digested with EcoRI and BamHI from pGFP-NleE_6A_ and ligated into pTrc99A. To create pNleB, *nleB* from pGFP-NleB was digested with EcoRI and BamHI and ligated into pTrc99A.

**Anti-GFP western blotting.** HeLa cells were transfected with derivatives of pEGFP-C2 and incubated for 16 h. Cell lysis was performed by incubating cells in cold lysis buffer (50 mM Tris-HCl pH 8.0, 150 mM NaCl, 5 mM EDTA, 1% NP-40) on ice for 5 min before collecting lysate and incubating on ice for a further 10 min. Cell debris was pelleted and equal volumes of supernatant were collected for SDS-PAGE. Proteins transferred to nitrocellulose membranes were probed with rabbit polyclonal anti-GFP (Abcam) diluted 1:5000.

**Cell culture.** HeLa cells were maintained in DMEM supplemented with 10% FBS and 1% HEPES buffer in 5% CO_2_ at 37°C. 16 to 24 h before either transfection or infection, cells were seeded onto 12 mm glass cover slips (Menzel-Glaser, Braunschweig, Germany) within 24-well tissue culture trays (Sarstedt, Leicestershire, UK) at a density of 10^5^ cells per well. Caco-2 cells were purchased from the European Collection of Cell Cultures at passage number 46. Cells were cultured in T75 cm^2^ tissue culture flasks (Triple Red, U.K) in DMEM supplemented with 20% vol/vol heat inactivated foetal calf serum 100 U ml^-1^ penicillin, 100 μg ml^-1^ streptomycin, 2 mM L-glutamine and 1% non-essential amino acids (Sigma UK) and maintained at 37^o^C in a humidified 5% CO_2_ atmosphere. Cells were split 1:5 after cell monolayers had reached ~80-90% confluence. For experiments cells were used between passages 48-59, seeded at high density into 12 well plates (Triple Red, UK) and incubated until 2 days post confluent.

**HeLa and Caco-2 infection protocol.** For infection of HeLa cells, EPEC derivatives were grown in Luria broth (LB) for 8 h before being subcultured into DMEM and incubated stationary for approximately 16 h at 37°C with 5% CO_2_. 10 μl of this culture, OD_600_ ~ 1.0 nm, was used to infect each well for 2 h. The media was replaced after 90 min with DMEM with or without 20 ng/ml TNF-α for the final 30 min. For Caco-2 cells, 2 h prior to infection, the medium from Caco-2 cell cultures was removed and monolayers washed with DMEM supplemented with 2 mM L-glutamine and 1% non-essential amino acids. Monolayers were subsequently incubated in DMEM at 37^o^C in a 5% CO_2_ atmosphere until the end of the experiment. To activate bacteria, overnight cultures of EPEC were inoculated 1:40 into DMEM and incubated for 2 hours in a 5% CO_2_ atmosphere at 37^o^C. A sufficient volume of activated bacteria was inoculated into cell cultures to reach a multiplicity of infection (MOI) of ~10:1. The MOI and infection times were tested empirically using wild type EPEC to determine which infection conditions yielded ~ 90%-100% cell viability at the end of the infection assay. Cell viability was assessed visually by trypan blue dye exclusion and when appropriate, dye exclusion assays were routinely carried out at the end of experiments. After 4 hours of infection Caco-2 monolayers were carefully washed twice with DMEM to remove non-adherent bacteria. Monolayers were then incubated in DMEM supplemented with 100 μg/ml gentamicin for 2 hours in order to kill adherent bacteria. After incubation with antibiotics monolayers were carefully washed twice with DMEM to remove bacterial debris. Cells were then incubated in DMEM supplemented with 50 μg/ml gentamicin with or without 5 ng/ml IL-1β as appropriate. For mRNA analysis monolayers were incubated for 3 hours and for analysis of IL-8 secretion monolayers were incubated for 24 hours.

**RNA extraction, cDNA synthesis and real-time PCR analysis.** Total cellular RNA was extracted using Trizol (Invitrogen UK) according to the manufacturer’s instructions. DNase treatment and cDNA synthesis was performed as previously described [1]. The Rotor-Gene 6000 real-time thermal cycler (Qiagen) was used to measure gene expression. The relative expression of *IL8* was determined using the comparative quantification method included in the Rotor-Gene 1.7 software (Qiagen). Ribosomal phosphoprotein P0 (RPLP0) served as a housekeeping gene for RNA extracted from Caco-2 cells since it has been shown that this gene is stably expressed in this cell line [2]. PCR reactions were carried out in a 20 μl volume comprising 10 μl of 2x SYBR Green JumpStart Taq ReadyMix (Sigma UK), 0.5 μM of each sense and antisense primer in a total volume of 2 μl, 6 μl of H_2_0 and 2 μl of cDNA. Cycle parameters comprised one hot start step of 5 minutes at 95°C and 40 cycles comprising 95°C for 15 seconds, 60°C for 20 seconds and 72°C for 25 seconds. PCR product specificity was confirmed by melt curve analysis and agarose gel electrophoresis. The gene specific primers for RPLP0 and *IL8* have previously been described. RPLP0: sense 5′-GCAATGTTGCCAGTGTCTG-3′ antisense 5′-GCCTTGACCTTTTCAGCAA-3′, IL-8: sense 5'-ATGACTTCCAAGCTGGCCGTGGC-3', antisense 5'-TCTCAGCCCTCTTCAAAAACTTC-3' [3].

**Supplementary references**

1. Schuller S, Lucas M, Kaper JB, Giron JA, Phillips AD (2009) The ex vivo response of human intestinal mucosa to enteropathogenic Escherichia coli infection. Cell Microbiol 11: 521-530.

2. Dydensborg AB, Herring E, Auclair J, Tremblay E, Beaulieu JF (2006) Normalizing genes for quantitative RT-PCR in differentiating human intestinal epithelial cells and adenocarcinomas of the colon. Am J Physiol Gastrointest Liver Physiol 290: G1067-1074.

3. Wehkamp J, Harder J, Weichenthal M, Schwab M, Schaffeler E, et al. (2004) NOD2 (CARD15) mutations in Crohn's disease are associated with diminished mucosal alpha-defensin expression. Gut 53: 1658-1664.

**Table S1. Oligonucleotide primers used in this study for vector construction.**

| Primer | Sequence 5’-3’ |
| --- | --- |
| NleE_F_ | aagaattcatgattaatcctgttacta |
| NleE_F-pTrc_ | catgccatggttaatcctgttac |
| NleE188-224_F_ | aagaattcaaatctgaaatgattatcgc |
| NleE_R_ | gggatccgtctactcaattttagaaag |
| NleE1-188_R-C2_ | gtggatccctatactgcctttaactctgg |
| NleE1-200_R-C2_ | cggatcccttctcccatttcacgtgcg |
| NleE1-208_R-C2_ | cggatcccttccccaggcatgtagct |
| NleE1-214_R-C2_ | cggatccctttcatataactgtctatttcc |
| NleE_I209A-R_ | gggatccgtctactcaattttagaaagtttattatttatgtatttcatataactgtccgc ttc |
| NleE_D210A-R_ | gggatccgtctactcaattttagaaagtttattatttatgtatttcatataactcgctat ttc |
| NleE_S211A-R_ | gggatccgtctactcaattttagaaagtttattatttatgtatttcatatacgcgtctat ttc |
| NleE_Y212A-R_ | gggatccgtctactcaattttagaaagtttattatttatgtatttcatcgcactgtctatttc |
| NleE_M213A-R_ | gggatccgtctactcaattttagaaagtttattatttatgtatttcgcataactgtctat ttc |
| NleE_K214A-R_ | ggtcgacctactcaattttagaaagtttattatttatgtacgccat |
| NleE_ΔIDSYMK-R_ | cgggatccgtctactcaattttagaaagtttattatttatgtattccccaggcatgta gctaaagatttctcccatttcacg |
| NleE_CR-F_ | aagaattcatgattaatcccatcacaa |
| NleE_CR-R_ | gcggtggatcccctaagtgcgtgaccttc |
| OspZ_F_ | aagaattaatgattagtcccatcaaga |
| OspZ_SF2a-R_ | gcggtggatcccttaatagactttaatctctg |
| OspZ_SF6-R_ | gcggtggatcccttaagtaacaggcattcg |
| OspZ_SB-R_ | gcggtggatcccttaagtaacagacattcg |
| OspZ208_R_ | ttcctctgatgcataac |
| OspZ_ΔIDSYIK-R_ | cggatcccttaagtaacaggcattcgagccttgatttgagaaaacctgtcattcatttgtgcctctgatgcataac |
| NleE183_R_ | gatttaatagactttaactctggtaactcataatttaattcc |
| OspZ184_F_ | aattaaattatgagttaccagagttaaagtctattaaatc |
| OspZ183_R_ | gattttactgcctttaactctggcgactcatctc |
| NleE184_F_ | tgttaagagatgagtcgccagagttaaaggcagtaaaatc |
| NleE6A_R_ | cgcggatccctactcaattttagaaagtttattatttatgtatgccgcagcagcggctgcttccccaggc |
| NleH1_F_ | aagaattcatgttatcgccctcttcta |
| NleH1_R_ | gggatccgtttatatcttacttaatacta |
| NleH2_F_ | aagaattcatgttatcgccatattctg |
| NleH2_R_ | gggatccgtctaaattttacttaatacc |
| NleB_F_ | aagaattcatgttatcttcattaaatgtc |
| NleB_R_ | gggatccttaccatgaactgctgg |
| NleD_F_ | aagaattcatgcgccctacgtccctc |
| NleD_R_ | gcggtggatcccctaaagcaatggatgcagtc |
| NleE6A_R-pTrc_ | cgcctcgacctactcaattttagaaagtttattatttatg |
